# Novel truncating Desmin mutation Arg150Stop disrupts structural integrity and cellular homeostasis by formation of persistent aggregate-like structure

**DOI:** 10.1038/s41420-026-03184-x

**Published:** 2026-06-05

**Authors:** Sukanya Mitra, Trisha Ghosh, Arun Kumar Mishra, Shamita Sanga, Sai Gayathri R, Mahua Maulik, Moulinath Acharya

**Affiliations:** 1 BRIC-National Institute of Biomedical Genomics, Kalyani, India; 2 Regional Centre of Biotechnology, Faridabad, India; 3 Present Address: CSIR-Institute of Genomics and Integrative Biology, New Delhi, India

**Keywords:** Intermediate filaments, Functional genomics

## Abstract

Desminopathies are a heterogeneous group of myofibrillar myopathies defined by the presence of desmin-positive aggregates that compromise cytoskeletal integrity in skeletal and cardiac muscle. Although desmin knockout models and several truncating mutations typically result in a functional null phenotype without inclusion body formation, the molecular consequences of specific stop-gain variants remain poorly understood. In this study, we investigated the pathogenic mechanism of a novel Desmin (DES) nonsense mutation, NM_001927.4:c.448 C > T; p.(Arg150Stop), previously identified in an Indian patient with congenital myopathy. This premature stop codon lies within the Linker 1 A domain and is predicted to generate a truncated protein lacking the C-terminal tail. To delineate its functional consequences, we used two complementary experimental approaches: transient overexpression of the Arg150Stop mutant in skeletal and cardiac myocytes, and a CRISPR/Cas9-engineered homozygous Arg150stop cardiomyocyte line (Des^tr^-CRISPR). Both models consistently revealed the formation of persistent aggregate-like structures, in striking contrast to desmin knockout systems that do not generate inclusions. These aggregate-like structures disrupted actin filament organization, impaired filament bundling, and induced organelle mislocalization. Biochemical analysis indicated that the aggregates were resistant to proteasomal degradation, yet they were partially cleared by autophagy, underscoring a role for protein quality control pathways in modulating disease severity. Importantly, the Des^tr^-CRISPR line demonstrated aggregate-driven pathology at endogenous levels, confirming that this mutation acts through a toxic gain-of-function mechanism rather than simple loss of desmin function. Our findings establish the Arg150Stop variant as a mechanistically distinct truncating mutation that generates aggregation-prone protein rather than a null state. By reproducing hallmark features of desminopathy in a physiologically relevant human cell models, this work not only broadens the known pathogenic spectrum of DES variants but also highlights aggregate formation as a central driver of cellular dysfunction and a promising therapeutic target in desminopathies.

**Figure:**
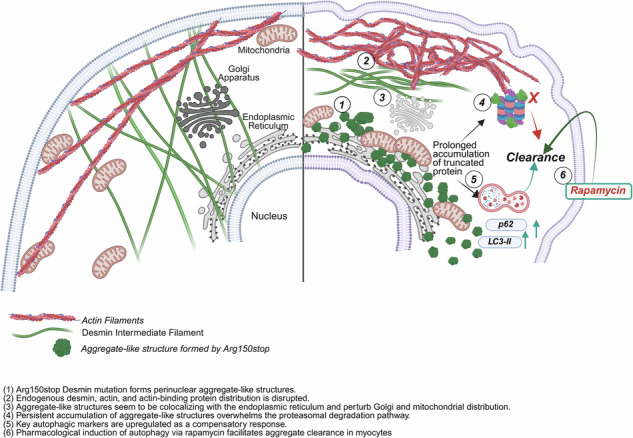

## Introduction

Desmin, a 53-kDa type III intermediate filament (IF) protein, is the major muscle-specific IF expressed in cardiac, skeletal, and smooth muscle, serving as a central architectural and functional element [1, 2]. IFs such as desmin provide mechanical resilience and structural integrity through a robust cytoplasmic network [1]. Its exclusive expression in muscle underlines why desmin dysfunction profoundly disrupts muscle physiology [3]. Structurally, desmin follows the tripartite IF organization with a conserved α-helical coiled-coil “rod” flanked by variable head and tail domains [4]. The N-terminal head is essential for filament assembly, while the C-terminal tail governs network organization and protein interactions [5]. Within muscle fibers, desmin monomers dimerize and assemble into a three-dimensional filamentous network around Z-discs. This scaffold interconnects adjacent myofibrils and anchors the contractile apparatus to the sarcolemma (costameres), extracellular matrix, intercalated discs, and organelles such as nuclei and mitochondria [6, 7]. Thus, desmin ensures mechanical stability, preserves cell architecture, regulates organelle positioning, and contributes to protein quality control [1, 6].

Mutations in *DES* gene cause desminopathies, clinically heterogenous myofibrillar myopathies that are often accompanied by dilated cardiomyopathy [3]. It is characterized by skeletal muscle weakness, which is slowly progressive and predominant in the distal rather than proximal muscles and in lower limb than in upper limb [8]. A defining pathological hallmark of desminopathies is the abnormal accumulation of desmin within muscle fibres, forming amorphous eosinophilic deposits that appear as granular or granulofilamentous material upon electron microscopic examination [8]. Biochemical studies show that aggregated desmin adopts amyloid-like fibrils that are cytotoxic to muscle cells and severely disrupt sarcomeric organization [9]. Cellular protein quality control relies mainly on the ubiquitin–proteasome system (UPS), which degrades soluble misfolded proteins, and the autophagy–lysosome pathway, which clears defective organelles and larger protein aggregates. Although both pathways are essential for clearance of aggregation prone proteins, desmin-containing aggregates often overwhelms these pathways leading to impairment of proteasomal activity [10] and resistance to autophagic clearance [7], leading to their persistence and contributing to progressive myofibrillar degeneration.

Most pathogenic DES mutations cluster within the highly conserved α-helical rod domain, which is essential for filament assembly and cytoskeletal integrity. Single-point mutations in this region frequently disrupt polymerization, leading to intermediate filament collapse and cytoplasmic aggregates [11–15]. The majority of truncating mutations are located in the C-terminal region and trigger nonsense-mediated mRNA decay (NMD), resulting in a complete loss of desmin protein and a classical loss-of-function phenotype [6, 16–18]. Consistently, desmin-null mice and cell models also exhibit a complete absence of desmin, confirming that loss of the protein alone disrupts filament assembly and cytoskeletal integrity [19, 20]. However, these desmin-null models fail to fully recapitulate the disease phenotype associated with aggregation. There are also reports of truncating mutations in the linker 1 A region and 1B domain, such as p.K144X [16, 21] and p.L200fsX20 [16], but their effects on aggregation and the molecular mechanisms driving disease pathogenesis have not been characterized, leaving a gap in understanding the cellular consequences of such truncating variants. Along these lines, the molecular and cellular effects of the novel Arg150stop truncating mutation, previously reported from our lab [22] and located within the linker L1A a critical segment for filament flexibility and stability remain unexplored.

To address this, we employed two complementary experimental systems: transient overexpression of Arg150Stop desmin in skeletal myocytes and cardiomyocytes, and a heterozygous Des^tr^-CRISPR cardiomyocyte model. The Arg150Stop mutant cells exhibited perinuclear desmin-positive structures, disrupted cytoskeletal architecture, and mislocalized organelles, which were partially resolved upon autophagy activation. Similarly, the heterozygous Des^tr^-CRISPR model also exhibited aggregate-like structures. Taken together, these observations suggest that Arg150Stop behaves as a conditional gain-of-function mutation, distinct from other truncating DES variants that undergo NMD, justifying its detailed molecular characterization and highlighting the importance of cellular context in interpreting desminopathy mechanisms.

## Results

### *Arg150stop* desmin mutation leads to premature truncation and perinuclear aggregation in myocytes

**Fig. 1:**
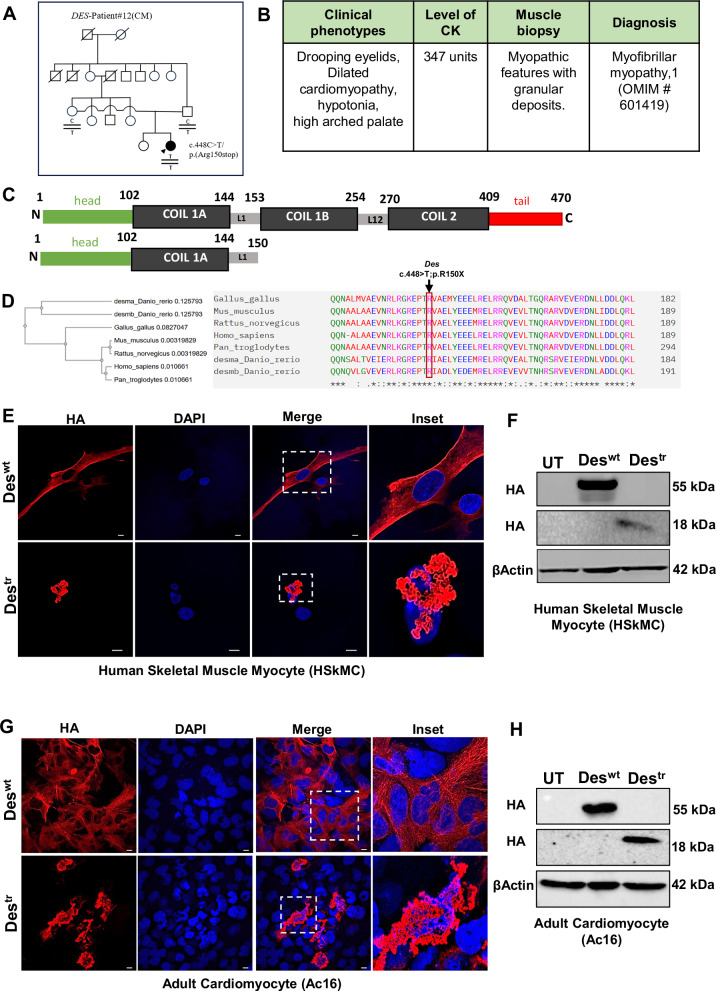
Characterization of the Arg150stop desmin mutation. **A** Segregation analysis confirming the presence of the Arg150stop desmin mutation in the proband (16). **B** Clinical features of the proband carrying the Arg150stop mutation. **C** Schematic representation of full-length desmin and the truncated protein formed due to Arg150stop mutation. **D** Multiple sequence alignment showing that Arginine (R) at position 150 is highly conserved across species. **E** Confocal microscopy images (60X) of human skeletal muscle myocytes demonstrating perinuclear aggregate-like structures formed by truncated Arg150stop Desmin (Scale bar 10 μm). **F** Western blot analysis confirming expression of the truncated 18 kDa protein in Human Skeletal Muscle Myocytes (HSkMCs). **G** Confocal microscopy images (60X) of Adult Cardiomyocytes showing perinuclear aggregate-like structures in truncated Arg150stop desmin expressing cells (Scale bar 10 μm). **H** Western blot analysis confirming the 18 kDa truncated protein in adult cardiomyocytes.

The stop gain mutation NM_001927.4: c.448 C > T; p. (Arg150Stop) in the *DES* gene, introduces a premature stop codon within exon 1. This homozygous mutation was found in a patient born to consanguineous parents and presented with hallmark clinical features of desminopathy, including early-onset hypotonia, dilated cardiomyopathy (DCM), and granular deposits observed in skeletal muscle biopsy [22] —features consistent with myofibrillar myopathy (MFM) (Fig. 1A, B). This C-to-T transition results in truncation of the desmin protein at the 150th amino acid (Arg150Stop), effectively eliminating the rod domain necessary for filament formation and cytoskeletal integration (Fig. 1C). Multiple sequence alignment using Clustal Omega revealed that the Arg150 residue is highly conserved across species, underscoring its functional importance in desmin structure and filament assembly (Fig. 1D).

To investigate the functional consequences of this mutation, we generated constructs encoding HA-tagged wild-type (Des^wt^) and mutant (Des^tr^) Desmin and overexpressed them in primary human skeletal myocytes (HsKMCs) and cardiomyocyte-derived AC16 cells. Immunofluorescence analysis demonstrated that cells expressing Des^wt^ formed an organized and filamentous desmin network extending from the nucleus to the cell periphery, indicating proper filament assembly (Fig. 1E, G). In contrast, cells expressing Des^tr^ exhibited a marked disruption in filament formation, instead showing intense accumulation of desmin-positive aggregate-like structures localized predominantly in the perinuclear region (Fig. 1E, G). Western blot analysis confirmed the expression of 18 kDa truncated desmin protein in Des^tr^ expressing cells (Fig. 1F, H). Our findings indicate that Arg150stop Desmin mutant results in a truncated protein that preferentially accumulates in the perinuclear region of both skeletal and cardiac muscle cells.

### Truncated Arg150stop desmin disrupts intermediate filament organization and alters cytoskeletal architecture in myocytes

**Fig. 2:**
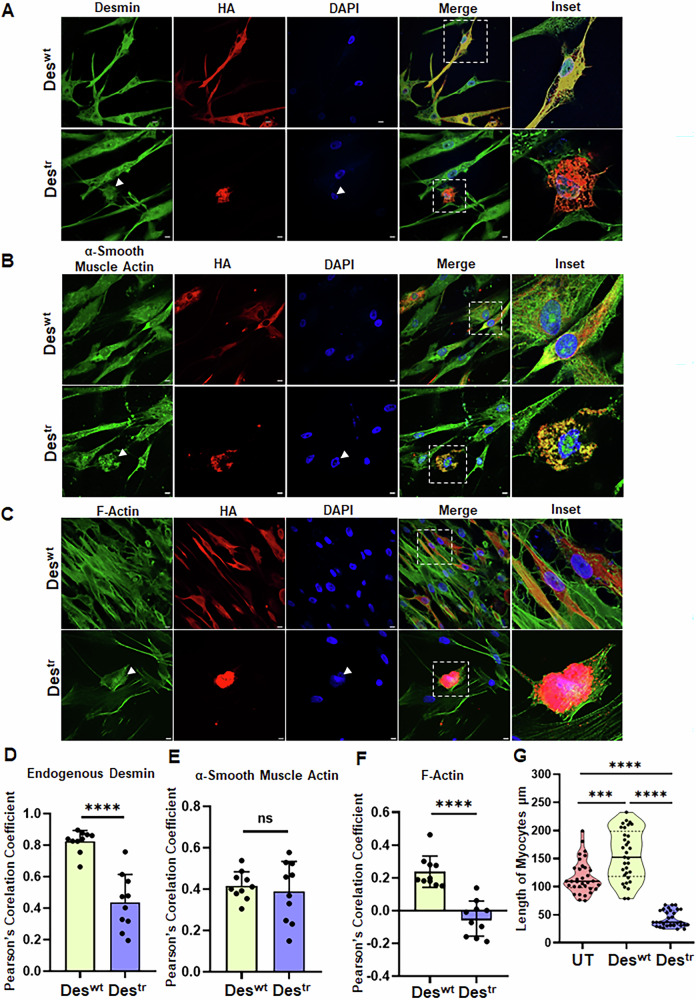
Arg150stop Desmin expression disrupts endogenous desmin organization and alters cellular morphology. **A** Confocal microscopy images (60X) transfected with Des^wt^ and Des^tr^, showing HA-tagged Desmin (red) and endogenous Desmin (green). Expression of Arg150stop leads to formation of aggregate-like structures and disruption of endogenous intermediate filament networks (Scale bar 10 μm). **B** Distribution of α-smooth muscle actin (green) in Des^wt^ and Des^tr^ transfected cells. Truncated desmin expression perturbs α-smooth muscle actin organization (Scale bar 10 μm). **C** Distribution of F-actin (green) in Des^wt^ and Des^tr^ transfected cells. Arg150stop expression alters F-actin organization (Scale bar 10 μm). **D** Pearson’s correlation coefficient analysis showing reduced colocalization between overexpressed HA-Desmin and endogenous desmin in Arg150stop transfected cells (*n* = 10, ****p < 0.0001). **E** Pearson’s correlation coefficient analysis of HA-Desmin with α-smooth muscle actin show no change in colocalization between overexpressed HA-Desmin and α-smooth muscle actin in truncated Arg150stop transfected cells (*n* = 10). **F** Pearson’s correlation coefficient analysis showing reduced colocalization between transiently expressed HA-Desmin and F-actin in Arg150stop transfected cells (*n* = 10, ****p < 0.0001). **G** Quantification of myocyte length showing altered cellular morphology upon truncated Arg150stop expression. Arg150stop transfected cells showed reduced myocyte length as compared to untransfected and wild-type expressing cells (*n* = 33, ***0.0003, **** *p* < 0.0001). All data are presented as the mean ± S.D.

Proper cytoskeletal organization is critical for myofibrillar architecture. Desmin forms a three-dimensional mesh around Z-discs, connecting them to the subsarcolemmal cytoskeleton, nuclei, and organelles to ensure mechanical integrity [1, 23]. As the truncated Arg150stop mutant accumulates perinuclearly (Fig. 1E, G), we hypothesized that this truncation may disrupt filament assembly and cytoskeletal organization. To investigate this, we transfected HsKMCs with either Des^wt^ or Des^tr^ and performed co-immunostaining for different cytoskeletal elements including endogenous desmin (Fig. 2A–F). In Des^wt^ transfected cells, endogenous Desmin exhibited the typical filamentous network similar to untransfected controls (Fig. 2A). Moreover, both the endogenous desmin and the transfected wild-type desmin showed a complete colocalization (*p* < 0.0001) in the transfected cells (Fig. 2D). However, expression of Arg150stop resulted in the collapse of this network, evident from loss of defined filamentous pattern, otherwise observed with endogenous desmin (Fig. 2A). Quantitative Pearson’s correlation analysis confirmed a significant reduction in colocalization between endogenous and truncated Desmin in Arg150stop desmin mutant cells compared to wild-type (Fig. 2D), indicating a dominant-negative effect of the mutant on filament integration.

We also observed alterations in gross cellular morphology, notably in cells forming aggregates (Fig. 2A–C, G). To assess whether the Arg150stop mutation impacts the broader cytoskeletal framework, we stained cells for α-smooth muscle actin (α-SMA) and filamentous actin (F-actin). Cells expressing Des^wt^ retained elongated morphology and showed well-organized, filamentous actin distribution consistent with normal cytoskeletal architecture (Fig. 2B, C). In contrast, truncated Arg150stop desmin mutant cells displayed disrupted actin networks, with shortened filament length and reduced colocalization with desmin (Fig. 2F, G). F-actin, which typically localizes at the cell periphery, showed poor spatial overlap with the Arg150stop desmin mutant. The mutant remained confined near the nucleus, further supporting impaired interaction between Desmin and actin filaments.

Additionally, we evaluated the distribution of actin-binding proteins, such as Plastin-1 (PLS1) and α-actinin-1 which are key regulators of actin filament cross-linking (Fig. S5A, B). Although none of these actin-binding proteins colocalized with wild-type or truncated Arg150stop desmin (Fig. S5C, D), their altered distribution in truncated Arg150stop desmin mutant cells implied morphological disarray. Collectively, these findings suggest that the truncated Arg150stop mutant forms aggregate-like structures that sequester endogenous desmin and perturb the structural alignment of actin filaments, ultimately compromising cytoskeletal integrity and myocyte morphology.

### Arg150stop desmin mutation disrupts organelle distribution and spatial organization in myocytes

**Fig. 3:**
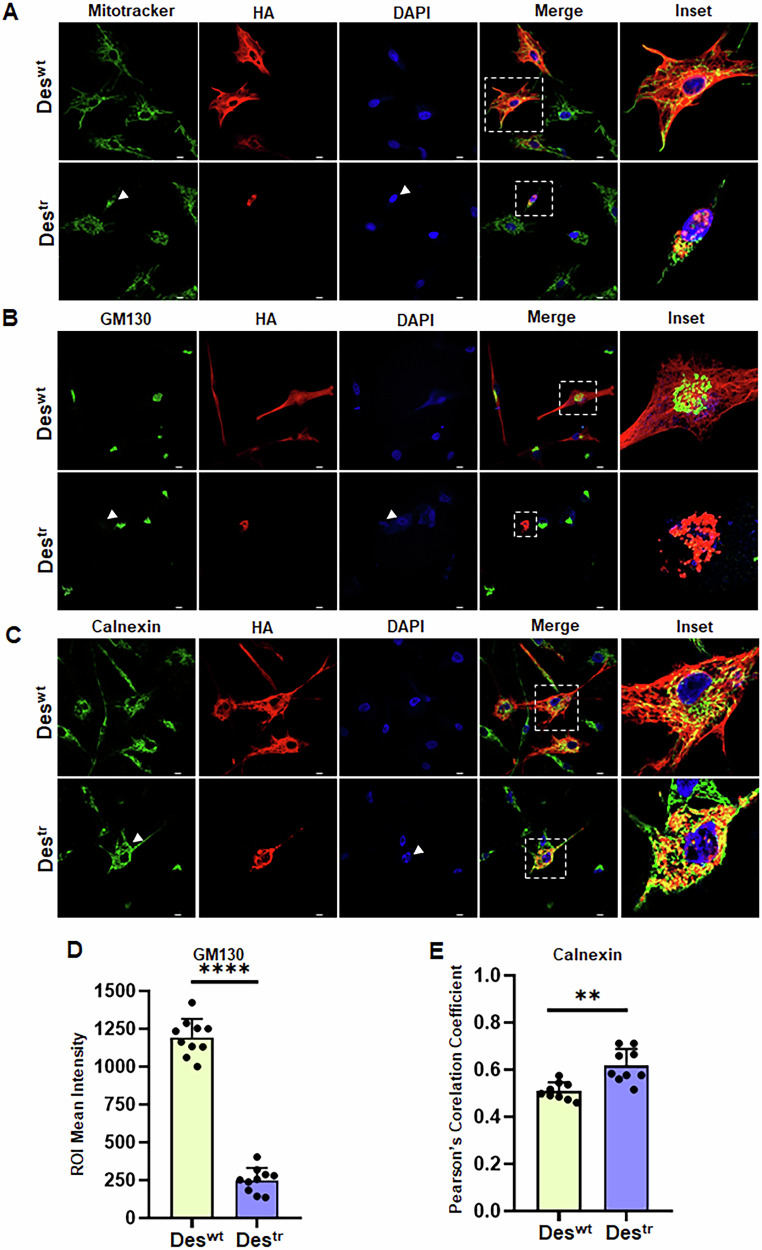
Arg150stop desmin aggregate-like structures disrupt organelle distribution. **A** Confocal microscopy images (60X) transfected with Des^wt^ and Des^tr^, showing HA-Desmin (red) and MitoTracker (green). In truncated Arg150stop cells expressing cells, mitochondria cluster around perinuclear aggregate-like structures (Scale bar 10 μm). **B** Confocal microscopy images (60X) transfected with Des^wt^ and Des^tr^, showing HA-Desmin (red) and GM130, a cis-Golgi marker (green). In wild-type cells, Golgi localizes at the nuclear periphery, whereas aggregate-like structures formed by Arg150stop form large perinuclear clumps with loss of Golgi signal (arrows) (Scale bar 10 μm). **C** Confocal microscopy images (60X) transfected with Des^wt^ and Des^tr^, showing HA-Desmin (red) co-immunostained with Calnexin (green). Aggregate-like structures formed by truncated Arg150stop appear to colocalize with Calnexin (Scale bar 10 μm). **D** Quantification of GM130 intensity showing a significant reduction in Golgi signal in truncated Arg150stop aggregate-harbouring cells (*n* = 10, ****p < 0.0001). **E** Pearson’s correlation coefficient analysis of HA-Desmin with Calnexin showing the truncated desmin colocalizing with Calnexin (*n* = 9, **p = 0.0052). All data are presented as the mean ± S.D.

Desmin has reportedly been shown to maintain intracellular architecture by anchoring and spatially organizing key organelles such as mitochondria, the Golgi, and the sarcoplasmic reticulum [23]. To investigate whether the truncated Arg150stop disrupts this organization, we examined mitochondrial, Golgi, and ER markers in transfected myocytes. In cells transfected with Des^tr^, mitochondrial distribution was notably altered compared to both Des^wt^ and untransfected controls. Mitochondria are evenly dispersed along the filamentous axis of the cell. The cells overexpressing Arg150stop showed distribution of mitochondria clustered abnormally near the perinuclear aggregates of the truncated desmin (Fig. 3A). Further, immunostaining with Golgi markers GM130 (cis-Golgi) (Fig. 3B, D), GCC2 (trans-Golgi) (Fig. S6), and p230 (trans-Golgi) (Fig. S6) revealed a significant reduction in Golgi signal intensity in truncated Arg150stop desmin mutant cells, suggesting impaired Golgi maintenance or positioning. Interestingly, co-localization analysis with Calnexin, the ER resident protein, indicated that the Arg150stop aggregates partially overlap with ER structures (Fig. 3C, E), raising the possibility of ER entrapment. Collectively, these findings suggest that the Arg150stop Desmin mutant disrupts the intermediate filament network, thereby altering the spatial distribution and potentially the function of critical organelles in myocytes.

### Truncated Arg150stop desmin mutant forms persistent aggregate-like structures in cardiomyocytes

**Fig. 4:**
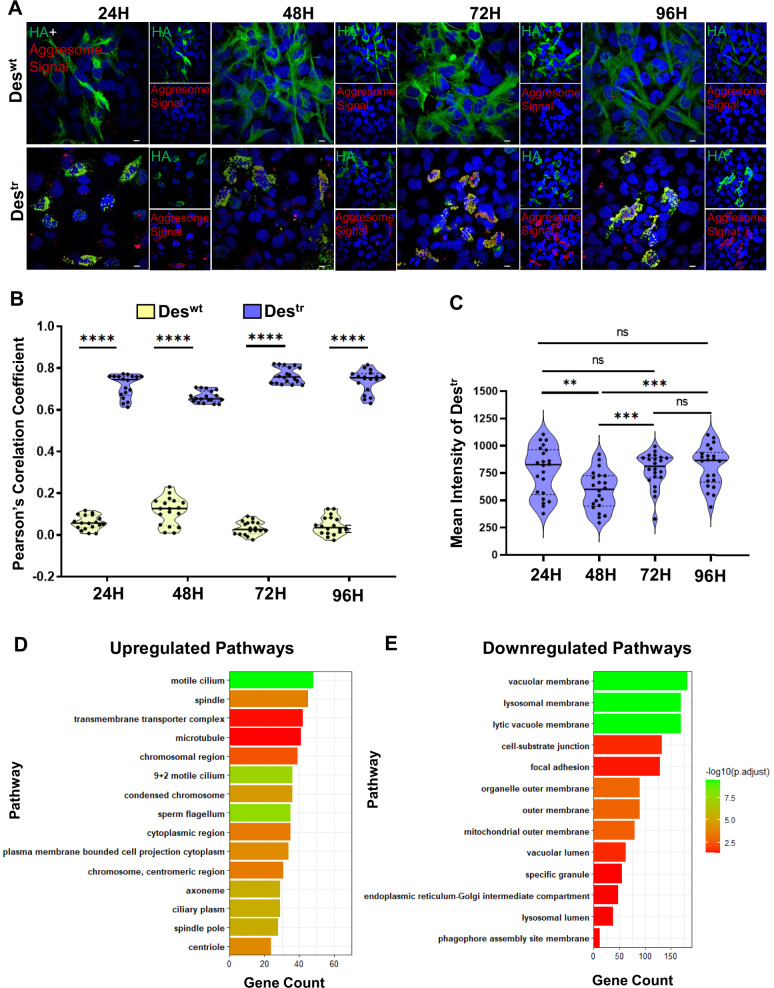
Persistent accumulation of Arg150stop aggregate-like structures is associated with transcriptomic alterations in cytoskeletal and degradative pathways. **A** Confocal microscopy images (60X) of cells transfected with Des^wt^ and Des^tr^, showing aggregate-like structures in truncated Arg150stop expressing cells detected by the aggresome detection assay. Overexpressed desmin is shown in the green channel, and the aggresome rotor dye in the Texas Red channel (Scale bar 10 μm). **B** Quantification of Pearson’s correlation coefficient shows increased colocalization of overexpressed desmin (green) with the aggresome rotor dye (red) in cells expressing Des^tr^ across all the four timepoints- 24 h,48 h,72 h and 96 h (*n* = 18, ****p < 0.0001). **C** Quantification of mean intensity of overexpressed desmin (green) reveals that aggregate-like structures formed by truncated Arg150stop persist without reduction over time (*n* = 22, **p = 0.0050[24 v/s48], ***p = 0.0005[48 v/s72], ***0.0002[48 v/s96]). **D** Pathway enrichment analysis revealed significant upregulation of microtubule-associated pathways in truncated Arg150stop mutant cells relative to wild-type cells. **E** Pathway enrichment analysis revealed significant downregulation of lysosomal-associated pathways in truncated Arg150stop mutant cells relative to wild-type cells. All data are presented as the mean ± S.D.

Several missense mutations in the *DES* gene have been previously reported to result in desmin misfolding and formation of intracellular aggregates or aggregate-like structures, which is a hallmark of desmin-related myopathies [11–15]. To determine whether the truncated Arg150stop mutant similarly forms aggregate-like structures, we transfected AC16 cardiomyocytes with Des^tr^ and stained them using a red fluorescent molecular rotor dye specific for aggregated proteins. We observed prominent co-localizing foci between truncated Arg150stop and the aggresomal dye in transfected cells, which were absent in cells expressing Des^wt^ (Fig. 4A). This finding was supported by a statistically significant increase in the Pearson’s correlation coefficient (*p* < 0.0001) for colocalization between the mutant desmin and aggresome-specific dye (Fig. 4B).

To assess the temporal dynamics of aggregate formation and clearance, we examined transfected cells at 24-, 48-, 72- and 96-h post-transfection. The aggregate-like structures were persistent throughout the time course, suggesting that these misfolded structures are not efficiently cleared (Fig. 4C). These observations indicate that the truncated Arg150stop mutant promotes stable aggregate-like structures in cardiomyocytes, highlighting a defect in the intrinsic protein quality control pathways responsible for maintaining desmin proteostasis. Immunoblot analysis at different time points under basal conditions showed a decreasing trend in Arg150stop expression over time, but these changes were not statistically significant, indicating that the mutant protein persists as aggregate-like structures (Fig. S7). RNA-sequencing followed by GO-Cellular Component pathway enrichment analysis revealed significant upregulation of microtubule-associated pathways (Fig. 4D). In mammalian cells, misfolded or aggregated proteins are transported along microtubules via dynein-mediated retrograde trafficking toward perinuclear aggresome-like structures, facilitating their spatial sequestration and potential clearance [24–26]. In contrast, several membrane-associated compartments were significantly downregulated, including vacuolar and lysosomal membrane components, lysosomal lumen, and ER–Golgi intermediate compartment pathways (Fig. 4E). Collectively, these data indicate a transcriptional shift favouring cytoskeletal reorganization while concomitantly reducing expression of vesicular and lysosomal machinery involved in protein processing and degradation.

### p62/SQSTM1 is upregulated and associates with Arg150stop aggregates-like structures in cardiomyocytes

Aggregate-like structures often recruit p62/SQSTM1, a multifunctional ubiquitin-binding scaffold that links polyubiquitinated aggregates to autophagosomes for degradation [27, 28]. Elevated p62 levels have been reported in Desmin mutant models as a compensatory response to proteotoxic stress [29]. To assess whether this mechanism is activated in response to the truncated desmin mutant, we assessed p62 expression in cardiomyocytes transfected with Des^tr^.

**Fig. 5:**
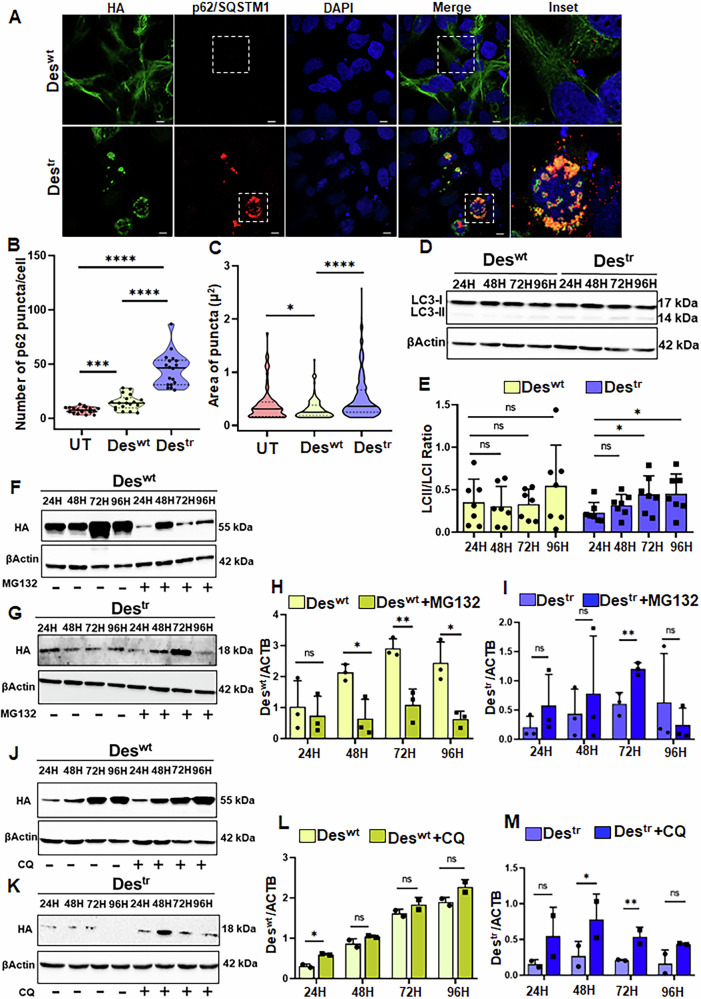
Arg150stop aggregate-like structures show p62 recruitment and altered LC3 expression as a compensatory response. **A** Confocal microscopy images (60X) of cells transfected with Des^wt^ and Des^tr^, shows that p62 colocalizes with truncated Arg150stop aggregates (Scale bar 10 μm). **B** Quantification of puncta number per cell indicate increased p62 recruitment upon expression of truncated Arg150stop (*n* = 18, ***p = 0.0003, ****p < 0.0001). **C** Quantification of puncta area indicate increased enlargement of p62 puncta upon expression of truncated Arg150stop (*p = 0.0181, ****p < 0.0001). **D** Western blot showing expression of LC-I and LC-II in wild-type and truncated Arg150stop desmin mutant expressing cells (Des^wt^ and Des^tr^). **E** Time-course analysis of LC3-II/I ratio in cells transfected with Des^wt^ and Des^tr^ depicts an increase of LCII/I ratio over time (*n* = 7, * *p* = 0.0412 [24 v/s72], * *p* = 0.0452 [24 v/s72]). **F** Western blot showing expression of wild-type desmin (Des^wt^) in the presence and absence of MG132 (5um, 6 h). **G** Western blot showing expression of truncated Arg150stop desmin (Des^tr^) in the presence and absence of MG132 (5um, 6 h). **H** Densitometric analysis of Des^wt^ in presence and absence of MG132 showed decreased expression of wild-type desmin levels upon MG132 treatment (*n* = 3). **I** Densitometric analysis of Des^tr^ expression in presence and absence of MG132 showed increasing trend in expression of truncated Arg150stop after MG132 treatment (*n* = 3). **J** Western blot showing expression of wild-type desmin (Des^wt^) in the presence and absence of Chloroquine (50um, 6 h). **K** Western blot showing expression of truncated Arg150stop desmin (Des^tr^) in the presence and absence of Chloroquine (50um, 6 h). **L** Densitometric analysis of Des^wt^ in presence and absence of Chloroquineshowed a increasing trend in expression of wild-type desmin levels upon Chloroquine treatment (*n* = 2). **M** Densitometric analysis of Des^tr^ expression in presence and absence of Chloroquine showed an increase in expression of truncated Arg150stop after Chloroquine treatment (*n* = 2). All data are presented as the mean ± S.D. CQ = Chloroquine.

A statistically significant increase in the number and area of p62-positive puncta was observed in truncated Arg150stop desmin mutant cells compared to cells transfected with full-length desmin (Des^wt^) (Fig. 5A–C). Notably, these p62-positive puncta were found in close spatial proximity to aggregate-like structures formed by truncated Arg150stop mutant, suggesting that p62 is actively recruited or sequestered with the misfolded protein structures. These observations indicate that p62/SQSTM1 accumulates around aggregate-like structures formed by truncated Arg150stop mutant as part of an adaptive proteostatic response. However, the continued presence of these aggregate-like structures despite p62 upregulation implies an insufficient or overwhelmed clearance system.

### Impaired clearance of truncated Arg150stop desmin promotes autophagic induction

Misfolded or truncated proteins are typically eliminated from the cytoplasm via tightly regulated degradation pathways such as the ubiquitin-proteasome system (UPS), the autophagy-lysosome pathway routes to maintain cellular proteostasis and prevent toxic protein accumulation [30–32]. We already observed that expression of truncated Arg150stop mutant showed a modest decline over time under basal conditions, and the absence of a significant reduction indicates inefficient clearance, consistent with the persistence of aggregate-like structures (Fig. S7). Given this persistence, we next investigated whether autophagy was activated as a compensatory mechanism in response to proteasomal insufficiency by assessing LC3-II/I levels. A time-dependent increase in the LC3-II/I ratio was observed in Des^tr^ transfected cells, particularly at 72 h and 96 h, whereas no such trend was evident in Des^wt^ transfected cells (Fig. 5D, E).

To investigate the clearance dynamics of the truncated Arg150stop desmin mutant, we selectively inhibited proteasomal degradation using MG132, a potent and reversible 26S proteasome inhibitor that blocks UPS-mediated degradation and promotes the accumulation of ubiquitinated proteins (36). We hypothesized that if truncated Arg150stop is primarily degraded via the proteasome, its intracellular levels would increase upon MG132 treatment. Consistent with this, MG132 exposure resulted in a trend toward increased accumulation of truncated Arg150stop desmin mutant across multiple time points, except at 96 h (Fig. 5F–I). Further, to assess the contribution of autophagy, cells were treated with chloroquine to inhibit autophagosome–lysosome fusion [33]. Chloroquine treatment led to a statistically significant increase in accumulation of truncated Arg150stop desmin mutant, indicating that blockade of autophagic flux enhances truncated protein buildup (Fig. 5J–M). Together, these findings suggest that proteasomal degradation alone is insufficient to efficiently clear aggregate-like structures formed by truncated Arg150stop desmin mutant. The elevated LC3-II/I ratio observed at 72 h and 96 h under basal conditions, along with the pronounced accumulation of truncated Arg150stop desmin mutant upon chloroquine treatment, supports the activation of autophagy as a compensatory, secondary clearance pathway.

### Induction of autophagy facilitates the partial clearance of R150 desmin mutant and restores cellular homeostasis in cardiomyocytes

**Fig. 6:**
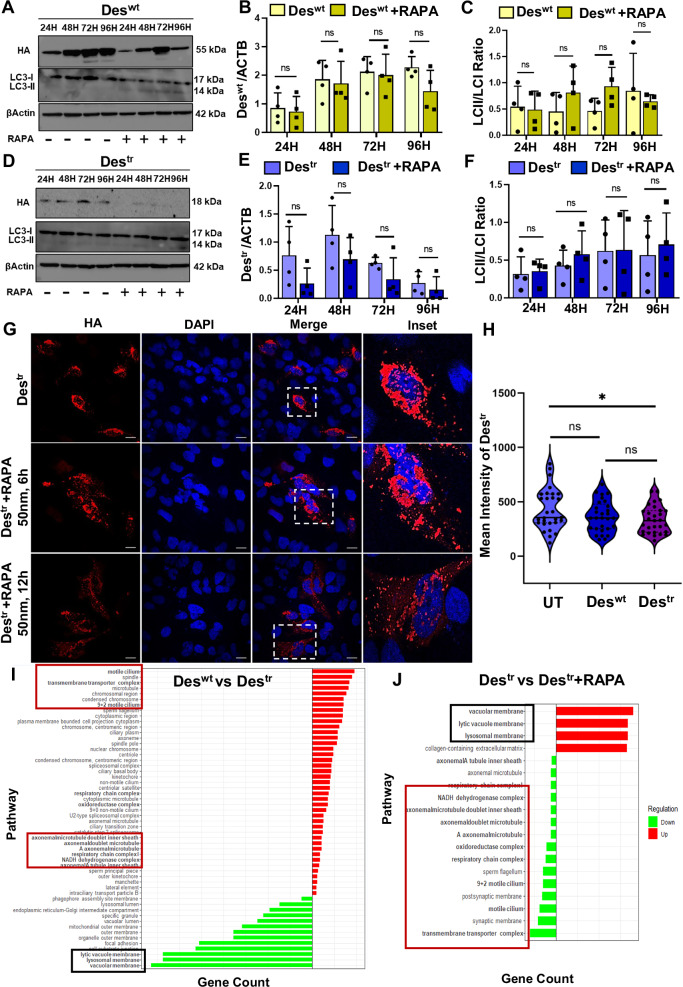
Rapamycin treatment reduces Arg150stop desmin aggregate-like structures and restores cellular homeostasis. **A** Western blot showing expression of wild-type desmin (Des^wt^) in the presence and absence of rapamycin (50 nm, 6 h). **B** Densitometric analysis of Des^wt^ in presence and absence of rapamycin showed no change in expression of wild-type desmin levels upon rapamycin treatment (*n* = 4). **C** LC3-II/I ratio in cells expressing Des^wt^ in presence or absence of rapamycin treatment (*n* = 4). **D** Western blot showing expression of truncated Arg150stop desmin (Des^tr^) in the presence and absence of rapamycin (50 nm, 6 h). **E** Densitometric analysis of Des^tr^ expression in the presence and absence of rapamycin showed reduced expression of truncated Arg150stop after rapamycin treatment (*n* = 4). **F** LC3-II/I ratio in cells expressing Des^tr^ in the presence or absence of rapamycin treatment (*n* = 4). **G** Confocal microscopy images (60X) of cells transfected with Des^tr^ show expression of truncated Arg150stop in presence or absence of rapamycin treatment for 6 h and 12 h (Scale bar 20 μm). **H** Quantification of mean intensity of overexpressed desmin (red) depicting reduction of Arg150Stop aggregates after rapamycin treatment (*n* = 30, *p = 0.0327). **I** Pathway enrichment analysis illustrating significantly upregulated and downregulated pathways in truncated Arg150stop mutant cells relative to wild-type cells. Differentially expressed gene sets were identified based on transcriptomic profiling and subjected to functional enrichment analysis to reveal cellular components altered between the two conditions. **J** Pathway enrichment analysis illustrating significantly upregulated and downregulated pathways in truncated Arg150stop mutant cells in the presence and absence of rapamycin. Upon rapamycin treatment, a reversal in transcriptomic profiling is observed (marked with brown and blue boxes). All data are presented as the mean ± S.D. RAPA=Rapamycin.

Autophagy, a critical homeostatic mechanism, degrades long-lived proteins and clears aggregated or misfolded proteins that evade proteasomal clearance [29, 34]. Since the truncated Arg150stop desmin mutant formed persistent aggregates, resisted UPS-mediated degradation, and showed elevated p62/SQSTM1 levels, we tested whether autophagy induction could facilitate its clearance. To evaluate this, we treated AC16 cardiomyocytes expressing either Des^wt^ or Des^tr^ with rapamycin, a well-characterized mTORC1 inhibitor and autophagy inducer, at four post-transfection time points: 24 h, 48 h, 72 h, and 96 h. Immunoblot analysis showed no change in Des^wt^ expression upon rapamycin treatment (Fig. 6A–C). In contrast, truncated Arg150stop mutant levels consistently decreased across all time points following treatment, suggesting enhanced autophagy-mediated clearance (Fig. 6D–F). However, LC3-II/I ratios remained unchanged in both wild-type and mutant conditions, regardless of rapamycin exposure (Fig. 6C, F). Immunofluorescence analysis revealed a reduction in Des^tr^ signal intensity following rapamycin treatment. A statistically significant decrease was observed after 12 h of treatment, whereas the reduction at 6 h did not reach significance (Fig. 5G, H). The decrease in both the number and area of p62-positive puncta following rapamycin treatment (Fig. S8) is consistent with increased autophagic flux and consumption of p62-bound aggregates.

Upon induction of autophagy with rapamycin, we observed a reversal of several dysregulated pathways. Pathways that were significantly upregulated in truncated Arg150stop desmin mutant cells, particularly those associated with microtubule organization, were downregulated following rapamycin treatment (Fig. 6I). This shift is consistent with the partial clearance of aggregate-like structures, potentially reducing the cellular reliance on microtubule-dependent trafficking mechanisms. Conversely, pathways that were previously downregulated, most notably those related to lysosomal membrane components, were upregulated upon rapamycin treatment (Fig. 6J). This suggests that enhancement of lysosomal machinery may contribute to improved aggregate clearance under autophagy-inducing conditions. Collectively, these results suggest that activation of autophagy through rapamycin treatment facilitates the clearance of truncated Arg150stop desmin mutant and may help restore cellular proteostasis in the context of desmin-related myopathies.

### Endogenous Arg150stop cardiomyocyte model mirrors overexpression-induced aggregation

**Fig. 7:**
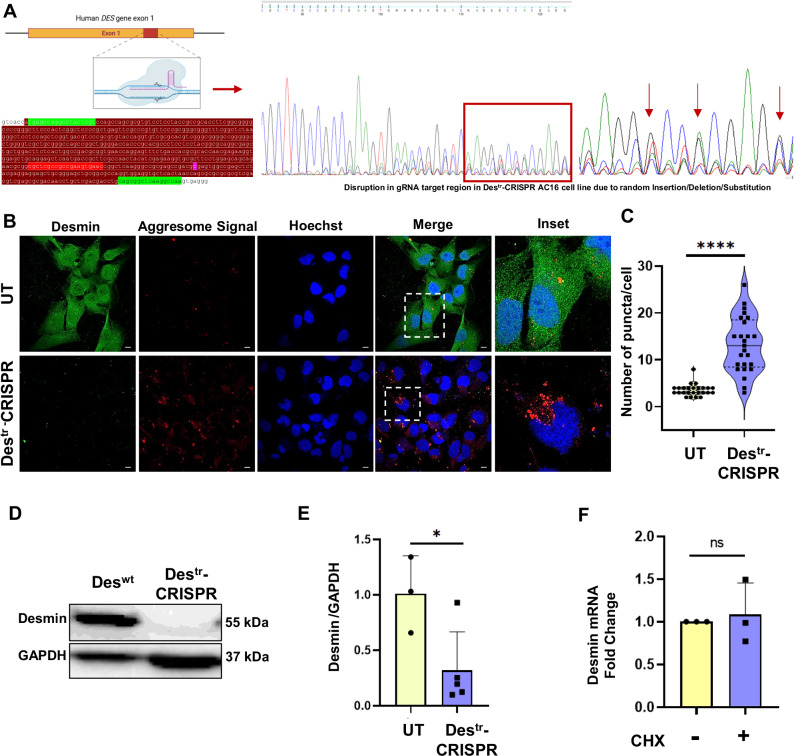
Characterization of Des^tr^-CRISPR cardiomyocyte model. **A** Schematic representation of the target region in exon 1 of the DES gene showing the gRNA binding site. Sanger sequencing traces confirm the successful introduction of insertions, deletions, and substitutions at the target locus. **B** Confocal microscopy images (60X) depicting increased aggresome rotor dye (red) signal in Des^tr^-CRISPR cell line (Scale bar 10 μm). **C** Quantification of aggresome puncta per cell depicting formation of aggregate-like structures in Des^tr^-CRISPR cell line (*n* = 25, ****p < 0.0001). **D** Western blot showing reduced endogenous desmin expression in Des^tr^-CRISPR cell line as compared to UT. **E** Densitometric analysis confirming decreased desmin levels in Des^tr^-CRISPR cell line (*n* = 5, *p = 0.0338). **F** Cycloheximide (CHX)-mediated inhibition of translation-dependent nonsense-mediated decay (NMD) did not result in a significant change in endogenous mRNA levels in Des^tr^-CRISPR cell line, suggesting that the mutant transcript is not targeted by NMD and may evade canonical RNA surveillance mechanisms (*n* = 3). All data are presented as the mean ± S.D. UT=Untransduced.

Complete loss of desmin abrogates the formation of desmin-positive inclusions, as knockout models fail to develop the aggregates characteristic of desmin-related myopathies [19, 20], supporting that inclusions arise from mutant or misfolded desmin rather than the absence of the protein. In this context, desmin deficiency may bypass aggregate nucleation compared to aggregate-forming conditions, which typically alter protein quality-control dynamics and autophagic response. To experimentally address this, we engineered the pathogenic variant Arg150stop in AC16 cardiomyocytes using CRISPR/Cas9, targeting exon 1 near the c.448 C > T transition to mimic the phenotype observed in our overexpression models (Fig. 7A). Quantitative confocal imaging revealed a significant increase in aggresome signal, as evident from elevated puncta numbers in Des^tr^-CRISPR compared with UT (Fig. 7B, C). Western blot analysis also confirmed a significant reduction in endogenous desmin levels (Fig. 7D, E). The detection of the truncated endogenous desmin protein was not possible with the antibody used, as it targets the C-terminal epitope absent in the truncated form. Premature truncating transcripts are typically subjected to nonsense-mediated mRNA decay (NMD). However, our immunofluorescence and immunoblot data suggested the presence of a truncated Arg150stop desmin mutant, prompting us to examine whether this transcript escapes NMD. To address this, AC16 cells were treated with cycloheximide (CHX), which inhibits translation elongation and thereby suppresses translation-dependent NMD by preventing recognition of premature termination codons [35–37]. In transcripts undergoing active NMD, CHX treatment is expected to stabilize the mRNA and increase its abundance. Notably, CHX treatment did not result in a significant increase in truncated desmin mRNA levels in the Des^tr^- CRISPR cell line, indicating that the truncated transcript is unlikely to be efficiently targeted by NMD under our experimental conditions (Fig. 7F). These findings demonstrate that the formation of persistent aggregate-like structures and their downstream effects on cardiomyocyte architecture and homeostasis are not restricted to overexpression systems. Through precise genome editing mimicking the pathogenic c.448 C > T variant in an endogenous context, we successfully recapitulated the aggregate-associated phenotype observed in the overexpression model, likely responsible for the granular deposits reported in the muscle biopsy of the patient [38].

## Discussion

### Novel pathogenic *DES* Arg150stop mutation as a model for Desminopathies

Desminopathies, particularly myofibrillar myopathies, are characterized by dense desmin-positive aggregates in skeletal and cardiac muscle, a feature consistently observed in cell and animal models [11–15, 29]. While many pathogenic DES mutations have elucidated how mutant desmin disrupts cellular architecture and homeostasis, most studies focus on single-point mutations or deletions, leaving truncating mutations largely unexplored. The novel DES Arg150stop mutation, identified in a 13-year-old girl from consanguineous parents, presents a distinct desminopathy phenotype with dilated cardiomyopathy and distal upper-limb weakness [22]. Muscle biopsy from the same patient published in another study revealed fiber-size variation, irregular Gömöri trichrome staining, an increase in NADH-TR activity, and ultrastructural abnormalities, including clustered lobulated nuclei and disrupted myofilaments with expanded Z-band aggregates, corroborating with histopathological features that are typical of desminopathies [38]. Despite these findings, the study did not elucidate the mechanistic basis of this truncating mutation in disease pathogenesis. Complementary immunohistochemistry and western blot analysis also demonstrated a complete absence of desmin expression. In contrast, our findings highlight that the C-to-T transition at nucleotide 448 produces a truncated desmin protein that retains the capacity to form aggregate-like structures, suggesting a toxic gain-of-function rather than simple loss of expression. Using both overexpression systems and a CRISPR/Cas9-engineered Arg150stop cardiomyocyte model, we demonstrate that this truncated protein is structurally compromised, aggregation-prone, bypasses non-sense mediated mRNA decay and is capable of disrupting the intermediate filament network—likely driving the observed myopathic phenotype. Thus, the Arg150stop variant reveals a more complex paradigm in which a truncating mutation can partially evade nonsense-mediated decay (NMD) and generate an aggregation-prone protein, an attribute typically linked to missense mutations, whereas most truncating variants result in loss-of-function through NMD. This suggests an overlap between loss- and gain-of-function mechanisms, refining current genotype–phenotype correlations in DES-associated disease. Therefore, given its reproducible aggregation behaviour, disruption of filament organization, and recapitulation of hallmark histopathological features in vitro, the Arg150stop mutation represents a powerful and physiologically relevant model for elucidating desminopathy mechanisms.

### Arg150stop disrupts intrinsic intermediate filament assembly and disorganizes myoskeletal organization and organellar positioning

Intermediate filaments (IFs) are essential for skeletal muscle integrity, interconnecting Z-discs, myofibrils, and linking them to the sarcolemma, nucleus, and mitochondria to maintain mechanical strength and sarcomere organization [39, 40]. The truncation at amino acid 150 abolishes the C-terminal domain, critical for filament bundling, higher-order organization, and docking of IF-associated proteins [41–43]. Loss of interactions with chaperones such as CRYAB and structural regulators like MTM1 likely promotes aberrant filament self-association and aggregate formation [39, 44, 45]. Moreover, desmin aggregates can impair actin filament organization [15, 46], as observed with actin-binding partners including Nebulin, Nebulette, and Z-disc–associated proteins plectin 1d, syncoilin, and synemin, whose interactions with desmin’s C-terminal are essential for actin network integrity [47–50]. Our immunocytochemistry data corroborate this, showing that truncated desmin disrupts actin filament organization around the nucleus, reflecting progressive loss of cytoskeletal coordination.

Disruption of desmin IFs impairs mitochondrial distribution and dynamics, compromising energy homeostasis and contractile function [51, 52]. Patient data also indicated mitochondrial displacement without overt morphological defects and a selective ~20% deficiency in complex IV activity [38]. These observations are further supported by our truncated Arg150stop mutant overexpressing cells, where RNA-sequencing analysis demonstrated downregulation of the mitochondrial outer membrane, suggesting compromised mitochondrial functional capacity. Additionally, we observed reduced Golgi fluorescence at the nuclear periphery in truncated Arg150stop mutant expressing cells. The Golgi, typically positioned via microtubule and actin attachments [53–56], may be affected due to disrupted actin architecture due to desmin aggregation. However, the precise mechanism underlying the reduced Golgi fluorescence intensity observed in truncated Arg150stop desmin mutant cells remains to be elucidated. Additionally, we observed downregulation of the ER-Golgi intermediate compartment pathway in our RNA-sequencing data, which might explain the accumulation at ER-exit sites and impairment of ER-Golgi transport, leading to retention of secretory cargos and delayed Golgi processing. Further, we also observed truncated desmin aggregates partially associated with the ER protein Calnexin, suggesting potential ER involvement. However, their impact on ER structure and dynamics remains unclear, as reported for another intermediate filament, vimentin [57]. The Arg150stop aggregates might also affect calcium flux, as supported by studies in Desma knockout zebrafish, which show unaltered muscular morphology but altered calcium flux [58]. Overall, while Arg150stop aggregates appear to influence organelle distribution and function, the underlying mechanisms require further investigation.

### Persistent aggregate-like structure formed by Arg150stop induces selective autophagy to clear off the truncated protein

We found that the truncated Arg150stop desmin mutant formed persistent aggregate-like structures of varying number, morphology, and distribution, often clustering perinuclearly. Aggresome detection assays confirmed their aggresomal association, though the full complement of associated proteins remains unknown. Misfolded proteins, such as this truncated variant, are inherently less stable due to improper folding and the exposure of hydrophobic residues, which predispose them to degradation via protein quality-control pathways [59]. While individual mutant molecules are unstable, their propensity to self-associate can drive the formation of persistent, aggregate-like structures that are resistant to clearance [60]. One such candidate, which we focused on in this study, is p62/SQSTM1, which facilitates aggresome formation and activates autophagy to protect cardiomyocytes from proteotoxic stress [28, 29]. Through its UBA domain, p62 recruits misfolded proteins; the PB1 domain drives oligomerization, and the LC3-interacting region (LIR) directs autophagic degradation, effectively linking the UPS and autophagy pathways [28]. p62 accumulation was also seen to be increased in patients harbouring desmin mutations carrying cardiomyopathic features [61]. As direct patient-derived samples corresponding to the variant investigated in our study were not available for biochemical validation, this association is drawn from previously published reports. Nevertheless, the concordance between our cellular observations and patient-based evidence strengthens the relevance of p62-mediated proteostatic responses in the context of desmin truncation.

Earlier studies suggest that inefficient UPS clearance could be potentially due to impairment of the 19S proteasome subunit in desminopathic mouse hearts [10]. As the 19S subunit channels substrates into the 20S core, dysfunction could contribute to desminopathy pathogenesis. Under stress, excess abnormal proteins can overwhelm the UPS, impair proteasome activity, and activate the autophagy–lysosome pathway to relieve proteotoxic stress [62]. In truncated Arg150stop mutants, the LC3-II/I ratio increased but remained insufficient to clear aggregates. Similar autophagy defects occur in LMNA^–/–^ mice, where mTORC1 hyperactivation impairs autophagy; rapamycin reduces mTORC1 signaling, improves muscle function, and extends lifespan [63]. Rapamycin treatment reduced aggregate-like structures formed by the truncated Arg150stop mutant, yet the LC3-II/I ratio remained unchanged. Although rapamycin activates autophagy via mTORC1 inhibition, LC3-II responses are context-dependent and may not always increase [64, 65]. Together, these findings suggest that while our results are consistent with enhanced autophagy, a more detailed analysis of flux would help strengthen this interpretation.

### Arg150stop desmin mutant influences global transcriptomic profile

Persistent accumulation of truncated Arg150stop desmin results in sustained proteotoxic stress and incomplete activation of selective autophagy. To understand how this structural disruption translates into broader cellular consequences, we performed pathway enrichment analysis comparing wild-type and truncated Arg150stop overexpressing cells, with and without rapamycin treatment. Truncated Arg150stop-expressing cells exhibited significant upregulation of microtubule-associated pathways, consistent with dynein-mediated retrograde transport of misfolded proteins toward perinuclear aggregate-like structures [25, 26]. Mitochondrial respiratory chain pathways are also enriched, possibly reflecting compensatory responses to mitochondrial redistribution and altered bioenergetic demand. Spliceosome-related pathways are similarly upregulated, suggesting altered RNA processing dynamics under chronic proteotoxic stress. Conversely, lysosomal membrane and ER–Golgi intermediate compartment (ERGIC) pathways are downregulated, indicating compromised degradative capacity and impaired early secretory trafficking. Given that ERGIC dynamics depend on intact cytoskeletal organization and microtubule-based transport, disruption of the desmin network and perinuclear aggregation may interfere with ER exit site function and vesicular trafficking.

Rapamycin treatment partially normalized this transcriptomic profile: microtubule- and mitochondrial-associated pathways were reduced, while lysosomal membrane pathways were restored, coinciding with partial aggregate clearance. Notably, spliceosome-related pathways did not show similar reversal, suggesting that RNA-processing alterations may represent a more stable adaptive response to chronic proteotoxic stress rather than a direct consequence of aggregate burden. Collectively, these findings support a model in which Arg150stop-induced aggregation disrupts trafficking and organelle homeostasis, triggers transcriptomic rewiring, and induces adaptive responses that are only partially rescued by autophagy activation.

Working with a transient overexpression system presents limitations, including variability in Des^tr^ expression upon rapamycin treatment. While the decreasing trend in expression of truncated Arg150stop was consistently observed, the magnitude varied across replicates, resulting in minimal statistical significance. This essentially reflects biological heterogeneity inherent to overexpression systems rather than an absence of the effect. This also highlights the need for robust cell and animal models to dissect how truncating desmin variants disrupt degradation pathways and drive disease pathogenesis. While C2C12-based models expressing mutant desmin [66] and ES cell systems with head-domain deletions [67] have been developed, a CRISPR-engineered muscle cell model harbouring truncating desmin mutation has never been reported. Our study addresses this gap by demonstrating aggregate-like structure formation and its functional implications in a Des^tr^-CRISPR cardiomyocyte line, corroborated by findings from our overexpression model. A key limitation of this model is that, due to desmin’s essential role in muscle cells, viable homozygous CRISPR clones harbouring truncated Arg150stop mutant could not be generated, as multiple attempts led to loss of cell viability. Consequently, all experiments were conducted in a heterozygous background. This aligns with previous studies showing that complete loss of desmin causes lethality in mouse and human models [19, 20]. Similarly, heterozygous patients and mice initially retain near-normal muscle structure, but cumulative proteotoxic stress from mutant desmin leads to late-onset pathology [6, 68]. This also highlights that the pathogenic mechanism of desmin-related myopathies, in which severe manifestations are frequently associated with autosomal recessive inheritance and are progressive, ultimately leading to fatal outcomes in affected patients. We also admit that our study is limited by its in vitro focus and does not yet fully resolve how organelle dysfunction and aggregate persistence translate into tissue-level pathology in vivo. Nevertheless, this system provides a physiologically relevant platform to deepen mechanistic insights into desminopathy pathogenesis and lays the groundwork for therapeutic exploration.

## Materials and methods

### Cell culture and cell lines

Primary human skeletal muscle cells (HSkMC; ScienCell Research Laboratories™) were cultured in Skeletal Muscle Cell Medium (SkMCM) supplemented with 5% fetal bovine serum (FBS; ScienCell), 1% Skeletal Muscle Cell Growth Supplement (SkMCGS), and 1% penicillin–streptomycin (P/S) antibiotic solution. Human cardiomyocyte cell line AC16 (ATCC®) was maintained in DMEM/F-12 medium supplemented with 12.5% FBS and 1% Antibiotic-Antimycotic Solution (100X). Human Embryonic Kidney (HEK293) cells (ATCC®) were cultured in DMEM supplemented with 10% FBS, Gibco and 1% Antibiotic-Antimycotic Solution (100X). All cells were cultured at 37 °C in a humidified atmosphere containing 5% CO₂.

### Gene cloning and recombinant vector construction

The coding sequence of full-length wild-type desmin (1440 bp; 470 amino acids) was PCR-amplified from cDNA synthesized from human skeletal muscle cells (HSkMCs) using gene-specific primers (Supplementary Table S1). The amplified product and the pCI mammalian expression vector containing an N-terminal HA tag were digested with EcoRI and XbaI restriction enzymes and ligated to generate the recombinant Desmin wild-type construct (Des^wt^). The Arg150Stop mutant construct (Des^tr^) was generated from the wild-type plasmid by site-directed mutagenesis (SDM) using complementary mutagenic primers (Supplementary Table S1) designed to introduce a premature stop codon at residue 150. Mutagenesis was performed according to the manufacturer’s instructions, followed by DpnI digestion to remove parental template DNA and transformation into competent cells. Positive colonies were selected for plasmid isolation using the QIAprep Spin Miniprep Kit (Qiagen). The integrity of all constructs and successful incorporation of the mutation was confirmed by Sanger sequencing using pCI vector–specific primers (Supplementary Table S1).

### Transient overexpression of wild-type and truncated desmin constructs in HSkMCs and AC16 cells

Recombinant plasmids encoding wild-type desmin (Des^wt^) and the Arg150Stop mutant variant (Des^tr^) were transiently transfected into primary human skeletal muscle cells (HSkMCs) and the AC16 cardiomyocyte cell line. Electroporation of HSkMCs was performed using the Neon Transfection System (Invitrogen) under the following conditions: voltage 1475 V, pulse width 20 ms, and 2 pulses. Transfection efficiency for both constructs in HSkMCs is shown in Fig. S1. For AC16 cells, transient transfection was carried out using FuGENE HD Transfection Reagent (Promega Corporation) following the manufacturer’s protocol.

### Lentivirus-mediated desmin Arg150stop mutant cell line generation

For Cas9 and sgRNA expression in AC16 cells, we used the lentiCRISPR v2 vector in combination with lentiviral packaging plasmids to generate and deliver the CRISPR/Cas9 system (Supplementary Table S2). sgRNA sequences were designed using the CRISPOR design tool (Supplementary Table S1), and cloning plus lentiviral production were performed as per established protocols with minor modifications [69]. sgRNAs were designed to target exon 1 of the DES gene in close proximity to the c.448 C > T transition site to enable precise CRISPR-mediated modification (Fig. S2). Briefly, plasmids were transfected into HEK293 cells, and after 72 h of incubation, culture supernatants were collected, and viral particles were concentrated using Lenti-X™ Concentrator (Takara). Viral titers were estimated with Lenti-X™ GoStix® (Takara), and the required volume for the desired multiplicity of infection (MOI) was calculated. AC16 cells were transduced with lentiviral particles at an MOI of 4 in the presence of polybrene (8 ng/mL) to facilitate viral attachment. After 24 h, the medium was replaced with fresh medium containing puromycin (2 µg/mL) and incubated for 48 h for selection of transduced cells, which were subsequently replated for downstream experiments. These Arg150stop mutant Desmin-expressing AC16 cells will hereafter be referred to as Des^tr^-CRISPR in the following sections.

### Chemical perturbation of protein quality control and mRNA surveillance pathways

To assess the involvement of proteasomal degradation and autophagy in the clearance of the Arg150stop desmin mutant, AC16 cells were treated with MG132, Chloroquine, and Rapamycin. All treatments were administered 6 h prior to the end of each post-transfection time point (24 h, 48 h, 72 h, and 96 h). MG132 was used at a final concentration of 5 μM for 6 h based on optimization studies (Fig. S3A). Chloroquine was used at a final concentration of 50 μM for 6 h following dose and time optimization (Fig. S3B, C). Rapamycin was applied at a final concentration of 50 nM for 6 h in all subsequent experiments following standardization (Fig. S3D, E).

To inhibit nonsense-mediated mRNA decay (NMD), cells were treated with cycloheximide at a final concentration of 50 µg/mL for 4 h prior to sample collection [70].

### Protein isolation and Western blotting

Protein lysates were prepared from both electroporated HSkMCs and transfected AC16 cells using lysis buffer containing 20 mM HEPES (pH 7.6), 20% glycerol, 100 mM NaCl, 1.5 mM MgCl₂, 0.2 mM EDTA, and 0.1% Triton X-100, supplemented with 1 mM DTT, 1 mM PMSF, and a protease inhibitor cocktail. Cell suspensions were sonicated on ice with three 10-s bursts. Total protein concentration was quantified using the Bradford assay to ensure equal sample loading. For western blotting, 50 μg of total protein was mixed with 2× Laemmli sample buffer, boiled at 95 °C for 5 min, resolved on 12% SDS-PAGE, and transferred to nitrocellulose membranes. Membranes were blocked in 5% non-fat milk prepared in TBST buffer for 1 h at room temperature. Blots were incubated with primary antibodies overnight at 4 °C, followed by HRP-conjugated secondary antibodies for 2 h at room temperature. Protein detection was performed using an enhanced chemiluminescence (ECL) detection system (Thermo Fisher Scientific). β-Actin was used as a loading control. The antibodies used are listed in Supplementary Table S3.

### Immunocytochemistry and confocal microscopy

Electroporated and transfected cells were fixed either with 4% paraformaldehyde for 15 min at room temperature or with cold acetone-methanol (1:1) for 5 min, depending on antibody-specific requirements. Following fixation, cells were permeabilized and blocked before primary antibody incubation. Coverslips were incubated with primary antibodies either overnight at 4 °C or for 1 h at room temperature, as per the respective antibody guidelines, followed by a 1-h incubation with secondary antibodies at room temperature. Nuclei were counterstained with DAPI for 5 min at room temperature. After washing, coverslips were mounted using ProLong™ Gold Antifade Mountant (Invitrogen), and imaging was performed using a Nikon Eclipse Ti2 confocal microscope. Confocal images were acquired using a 60× oil immersion objective, with excitation at 405, 488, 561, and 647 nm laser lines. For HSkMCs, glass coverslips were pre-coated with poly-L-lysine prior to electroporation. When mitochondrial staining was required, cells were incubated with 50 nM MitoTracker™ Red CMXRos in serum-free media for 30 min prior to fixation. The antibodies used are listed in Supplementary Table S4.

### Aggresomal detection assay

Formation of aggregate-like structures was monitored by using Aggresomal Detection Kit (Abcam) that incorporates a 488 nm excitable, red-shifted molecular rotor dye that enables the specific detection of misfolded and aggregated protein species sequestered within aggresomal and aggresomal-like inclusions in fixed and permeabilized cells. The fluorescence associated with aggregated proteins was validated using both positive control (5uM, 6 h MG132-treated cells) and negative control (Untreated as well as Untransfected cells) (Fig. S4).

AC16 cells were transfected with both Des^wt^ and Des^tr^ and further processing was done following the manufacturer’s protocol. The coverslips were incubated with HA primary antibody for visualization of recombinant desmin, and fluorescence signals corresponding to aggresomes were visualized using a standard Texas Red filter set.

### RNA-sequencing data processing and differential expression analysis

Raw paired-end FASTQ files from all samples were quality-checked using FastQC. Adapter sequences and low-quality bases were removed using fastp with automatic paired-end adapter detection. Post-trimming quality assessment was performed using FastQC, and results from FastQC and fastp were summarized using MultiQC. Trimmed reads were aligned to the human reference genome (GRCh38) using STAR, with gene annotations (GENCODE release 36). Alignment quality and RNA-seq–specific metrics were assessed using Qualimap, and multi-sample summaries were generated using Qualimap multi-bamqc. Gene-level read counts were generated from sorted BAM files using “featureCounts” and the resulting count matrix was imported into R for downstream analysis. Read counts were analyzed using DESeq2. Genes with low expression (total counts < 10 across all samples) were filtered prior to normalization, and size-factor normalization was applied, and differential expression was assessed using log2 fold-change thresholds calculated from normalized counts. Genes with |log2FC | ≥ 1 were classified as differentially expressed, while genes below this threshold were considered unchanged. Functional enrichment analyses of differentially expressed genes were performed using Gene Ontology Cellular Compartment pathway databases, with significance determined using Benjamini–Hochberg–adjusted *p*-values < 0.05.

### Image and statistical analysis

Regions of Interest (ROIs) were manually drawn for cells transfected with Des^wt^ and Des^tr^, and Pearson’s correlation coefficient was calculated using Nikon AR software (version 5.0) across the entire z-stack of each transfected cell. The number of puncta was quantified using the “Analyze Particles” function in Fiji (ImageJ) [71], and all densitometric analyses were also performed using Fiji. Statistical analysis was conducted using an unpaired t-test for all pairwise comparisons in GraphPad Prism (version 8.4.3), with *p* < 0.05 considered statistically significant.

All methods were performed in accordance with the relevant guidelines and regulations.

## Supplementary information

Supplementary Material

Supplementary Material

## Data Availability

The RNA-seq raw files supporting the conclusions of this article is available in the European Nucleotide Archive (ENA) under accession number PRJEB108980.

## References

[1] Paulin D, Li Z. Desmin: A major intermediate filament protein essential for the structural integrity and function of muscle. Exp Cell Res. 2004;301:1–7. 15501438 10.1016/j.yexcr.2004.08.004

[2] Hol EM, Capetanaki Y. Type III Intermediate filaments desmin, glial fibrillary acidic protein (GFAP), vimentin, and peripherin. Cold Spring Harb Perspect Biol. 2017;9:a021642. 29196434 10.1101/cshperspect.a021642 PMC5710105

[3] Joanne P, Hovhannisyan Y, Bencze M, Daher M-T, Parlakian A, Toutirais G et al. Absence of Desmin results in impaired adaptive response to mechanical overloading of skeletal muscle. Front Cell Dev Biol 2021; 9. 10.3389/fcell.2021.662133. 10.3389/fcell.2021.662133 PMC8320001 34336827

[4] Vlachakis D, Tsilafakis K, Kostavasili I, Kossida S, Mavroidis M. Unraveling Desmin’s head domain structure and function. Cells. 2024;13:603. 38607042 10.3390/cells13070603 PMC11012097

[5] Goldman RD, Cleland MM, Murthy P, Mahammad S, Kuczmarski ER. Inroads into the structure and function of intermediate filament networks. J Struct Biol. 2012;177:14–23. 22120848 10.1016/j.jsb.2011.11.017 PMC3269975

[6] Clemen CS, Herrmann H, Strelkov SV, Schröder R. Desminopathies: pathology and mechanisms. Acta Neuropathol. 2013;125:47–75. 23143191 10.1007/s00401-012-1057-6 PMC3535371

[7] McLendon PM, Robbins J. Desmin-related cardiomyopathy: an unfolding story. Am J Physiol - Heart Circ Physiol. 2011;301:H1220–H1228. 21784990 10.1152/ajpheart.00601.2011 PMC3197348

[8] Goldfarb LG, Vicart P, Goebel HH, Dalakas MC. Desmin myopathy. Brain. 2004;127:723–34. 14724127 10.1093/brain/awh033

[9] Kedia N, Arhzaouy K, Pittman SK, Sun Y, Batchelor M, Weihl CC, et al. Desmin forms toxic, seeding-competent amyloid aggregates that persist in muscle fibers. Proc Natl Acad Sci USA. 2019;116:16835–40. 31371504 10.1073/pnas.1908263116 PMC6708308

[10] Liu J, Chen Q, Huang W, Horak KM, Zheng H, Mestril R, et al. Impairment of the ubiquitin-proteasome system in desminopathy mouse hearts. FASEB J Publ Fed Am Soc Exp Biol. 2006;20:362–4. 10.1096/fj.05-4869fje 16371426

[11] Goudeau B, Rodrigues-Lima F, Fischer D, Casteras-Simon M, Sambuughin N, de Visser M, et al. Variable pathogenic potentials of mutations located in the desmin alpha-helical domain. Hum Mutat. 2006;27:906–13. 16865695 10.1002/humu.20351

[12] Bär H, Fischer D, Goudeau B, Kley RA, Clemen CS, Vicart P, et al. Pathogenic effects of a novel heterozygous R350P desmin mutation on the assembly of desmin intermediate filaments in vivo and in vitro. Hum Mol Genet. 2005;14:1251–60. 15800015 10.1093/hmg/ddi136

[13] Brodehl A, Dieding M, Klauke B, Dec E, Madaan S, Huang T, et al. The novel Desmin mutant p.A120D impairs filament formation, prevents intercalated disk localization, and causes sudden cardiac death. Circ Cardiovasc Genet. 2013;6:615–23. 24200904 10.1161/CIRCGENETICS.113.000103

[14] Goldfarb LG, Park K-Y, Cervenáková L, Gorokhova S, Lee H-S, Vasconcelos O, et al. Missense mutations in desmin associated with familial cardiac and skeletal myopathy. Nat Genet. 1998;19:402–3. 9697706 10.1038/1300

[15] Conover GM, Henderson SN, Gregorio CC. A myopathy-linked desmin mutation perturbs striated muscle actin filament architecture. Mol Biol Cell. 2009;20:834–45. 19005210 10.1091/mbc.E08-07-0753 PMC2633376

[16] Brodehl A, Gaertner-Rommel A, Milting H. Molecular insights into cardiomyopathies associated with desmin (DES) mutations. Biophys Rev. 2018;10:983–1006. 29926427 10.1007/s12551-018-0429-0 PMC6082305

[17] Eiber N, Fröb F, Schowalter M, Thiel C, Clemen CS, Schröder R, et al. Lack of Desmin in mice causes structural and functional disorders of neuromuscular junctions. Front Mol Neurosci. 2020;13:567084. 33192292 10.3389/fnmol.2020.567084 PMC7649329

[18] Durmuş H, Ayhan Ö, Çırak S, Deymeer F, Parman Y, Franke A, et al. Neuromuscular endplate pathology in recessive desminopathies: lessons from man and mice. Neurology. 2016;87:799–805. 27440146 10.1212/WNL.0000000000003004

[19] Li Z, Mericskay M, Agbulut O, Butler-Browne G, Carlsson L, Thornell LE, et al. Desmin is essential for the tensile strength and integrity of myofibrils but not for myogenic commitment, differentiation, and fusion of skeletal muscle. J Cell Biol. 1997;139:129–44. 9314534 10.1083/jcb.139.1.129 PMC2139820

[20] Milner DJ, Weitzer G, Tran D, Bradley A, Capetanaki Y. Disruption of muscle architecture and myocardial degeneration in mice lacking desmin. J Cell Biol. 1996;134:1255–70. 8794866 10.1083/jcb.134.5.1255 PMC2120972

[21] Wahbi K, Béhin A, Charron P, Dunand M, Richard P, Meune C, et al. High cardiovascular morbidity and mortality in myofibrillar myopathies due to DES gene mutations: a 10-year longitudinal study. Neuromuscul Disord. 2012;22:211–8. 22153487 10.1016/j.nmd.2011.10.019

[22] Sanga S, Ghosh A, Kumar K, Polavarapu K, Preethish-Kumar V, Vengalil S, et al. Whole-exome analyses of congenital muscular dystrophy and congenital myopathy patients from India reveal a wide spectrum of known and novel mutations. Eur J Neurol. 2021;28:992–1003. 33124102 10.1111/ene.14616

[23] Capetanaki Y, Bloch RJ, Kouloumenta A, Mavroidis M, Psarras S. Muscle intermediate filaments and their links to membranes and membranous organelles. Exp Cell Res. 2007;313:2063–76. 17509566 10.1016/j.yexcr.2007.03.033

[24] Kopito RR. Aggresomes, inclusion bodies and protein aggregation. Trends Cell Biol. 2000;10:524–30. 11121744 10.1016/s0962-8924(00)01852-3

[25] Characterization and Dynamics of Aggresome Formation by a Cytosolic Gfp-Chimera✪ | Journal of Cell Biology | Rockefeller University Press. https://rupress.org/jcb/article-abstract/146/6/1239/32005/Characterization-and-Dynamics-of-Aggresome?redirectedFrom=fulltext (accessed 15 Feb2026). 10.1083/jcb.146.6.1239 PMC2156127 10491388

[26] Johnston JA, Ward CL, Kopito RR. Aggresomes: a cellular response to misfolded proteins. J Cell Biol. 1998;143:1883–98. 9864362 10.1083/jcb.143.7.1883 PMC2175217

[27] Bjørkøy G, Lamark T, Brech A, Outzen H, Perander M, Overvatn A, et al. p62/SQSTM1 forms protein aggregates degraded by autophagy and has a protective effect on huntingtin-induced cell death. J Cell Biol. 2005;171:603–14. 16286508 10.1083/jcb.200507002 PMC2171557

[28] Seneci P. Chapter 5 - Selective disposal of insoluble protein aggregates: pick, transport and remove to cure. In: Seneci P (ed). Molecular targets in protein misfolding and neurodegenerative disease. Academic Press: San Diego, 2015. pp 183–227.

[29] Zheng Q, Su H, Ranek MJ, Wang X. Autophagy and p62 in Cardiac Proteinopathy. Circ Res. 2011;109:296–308. 21659648 10.1161/CIRCRESAHA.111.244707 PMC3142307

[30] Ciechanover A. Proteolysis: from the lysosome to ubiquitin and the proteasome. Nat Rev Mol Cell Biol. 2005;6:79–87. 15688069 10.1038/nrm1552

[31] Rubinsztein DC, Mariño G, Kroemer G. Autophagy and Aging. Cell. 2011;146:682–95. 21884931 10.1016/j.cell.2011.07.030

[32] Dikic I. Proteasomal and autophagic degradation systems. Annu Rev Biochem. 2017;86:193–224. 28460188 10.1146/annurev-biochem-061516-044908

[33] Mauthe M, Orhon I, Rocchi C, Zhou X, Luhr M, Hijlkema K-J, et al. Chloroquine inhibits autophagic flux by decreasing autophagosome-lysosome fusion. Autophagy. 2018;14:1435–55. 29940786 10.1080/15548627.2018.1474314 PMC6103682

[34] Mizushima N, Komatsu M. Autophagy: renovation of cells and tissues. Cell. 2011;147:728–41. 22078875 10.1016/j.cell.2011.10.026

[35] Lim KH, Han Z, Jeon HY, Kach J, Jing E, Weyn-Vanhentenryck S, et al. Antisense oligonucleotide modulation of non-productive alternative splicing upregulates gene expression. Nat Commun. 2020;11:3501. 32647108 10.1038/s41467-020-17093-9 PMC7347940

[36] Kim YJ, Nomakuchi T, Papaleonidopoulou F, Yang L, Zhang Q, Krainer AR. Gene-specific nonsense-mediated mRNA decay targeting for cystic fibrosis therapy. Nat Commun. 2022;13:2978. 35624092 10.1038/s41467-022-30668-y PMC9142507

[37] Salapa HE, Thibault PA, Libner CD, Ding Y, Clarke J-PWE, Denomy C, et al. hnRNP A1 dysfunction alters RNA splicing and drives neurodegeneration in multiple sclerosis (MS). Nat Commun. 2024;15:356. 38191621 10.1038/s41467-023-44658-1 PMC10774274

[38] Santhoshkumar R, Preethish-Kumar V, Polavarapu K, Reghunathan D, Chaudhari S, Satyamoorthy K, et al. A novel L1 linker mutation in Des resulted in total absence of protein. J Mol Neurosci. 2021;71:2468–73. 34106405 10.1007/s12031-021-01856-0

[39] Hnia K, Ramspacher C, Vermot J, Laporte J. Desmin in muscle and associated diseases: beyond the structural function. Cell Tissue Res. 2015;360:591–608. 25358400 10.1007/s00441-014-2016-4

[40] Capetanaki Y, Papathanasiou S, Diokmetzidou A, Vatsellas G, Tsikitis M. Desmin related disease: a matter of cell survival failure. Curr Opin Cell Biol. 2015;32:113–20. 25680090 10.1016/j.ceb.2015.01.004 PMC4365784

[41] Kaufmann E, Weber K, Geisler N. Intermediate filament forming ability of desmin derivatives lacking either the amino-terminal 67 or the carboxy-terminal 27 residues. J Mol Biol. 1985;185:733–42. 3903168 10.1016/0022-2836(85)90058-0

[42] Herrmann H, Hesse M, Reichenzeller M, Aebi U, Magin TM. Functional complexity of intermediate filament cytoskeletons: from structure to assembly to gene ablation. Int Rev Cytol. 2003;223:83–175. 12641211 10.1016/s0074-7696(05)23003-6

[43] Herrmann H, Aebi U. Intermediate filaments: structure and assembly. Cold Spring Harb Perspect Biol. 2016;8:a018242. 27803112 10.1101/cshperspect.a018242 PMC5088526

[44] Sharma S, Conover GM, Elliott JL, Der Perng M, Herrmann H, Quinlan RA. αB-crystallin is a sensor for assembly intermediates and for the subunit topology of desmin intermediate filaments. Cell Stress Chaperones. 2017;22:613–26. 28470624 10.1007/s12192-017-0788-7 PMC5465037

[45] Houck SA, Landsbury A, Clark JI, Quinlan RA. Multiple sites in αB-crystallin modulate its interactions with desmin filaments assembled in vitro. PloS One. 2011;6:e25859. 22096479 10.1371/journal.pone.0025859 PMC3212511

[46] Conover GM, Gregorio CC. The desmin coil 1B mutation K190A impairs nebulin Z-disc assembly and destabilizes actin thin filaments. J Cell Sci. 2011;124:3464–76. 21984811 10.1242/jcs.087080 PMC3196858

[47] Konieczny P, Fuchs P, Reipert S, Kunz WS, Zeöld A, Fischer I, et al. Myofiber integrity depends on desmin network targeting to Z-disks and costameres via distinct plectin isoforms. J Cell Biol. 2008;181:667–81. 18490514 10.1083/jcb.200711058 PMC2386106

[48] Bellin RM, Huiatt TW, Critchley DR, Robson RM. Synemin may function to directly link muscle cell intermediate filaments to both myofibrillar Z-lines and costameres. J Biol Chem. 2001;276:32330–7. 11418616 10.1074/jbc.M104005200

[49] Newey SE, Howman EV, Ponting CP, Benson MA, Nawrotzki R, Loh NY, et al. Syncoilin, a novel member of the intermediate filament superfamily that interacts with alpha-dystrobrevin in skeletal muscle. J Biol Chem. 2001;276:6645–55. 11053421 10.1074/jbc.M008305200

[50] Hernandez DA, Bennett CM, Dunina-Barkovskaya L, Wedig T, Capetanaki Y, Herrmann H, et al. Nebulette is a powerful cytolinker organizing desmin and actin in mouse hearts. Mol Biol Cell. 2016;27:3869–82. 27733623 10.1091/mbc.E16-04-0237 PMC5170609

[51] Milner DJ, Mavroidis M, Weisleder N, Capetanaki Y. Desmin cytoskeleton linked to muscle mitochondrial distribution and respiratory function. J Cell Biol. 2000;150:1283–98. 10995435 10.1083/jcb.150.6.1283 PMC2150713

[52] Winter L, Wittig I, Peeva V, Eggers B, Heidler J, Chevessier F, et al. Mutant desmin substantially perturbs mitochondrial morphology, function and maintenance in skeletal muscle tissue. Acta Neuropathol. 2016;132:453–73. 27393313 10.1007/s00401-016-1592-7 PMC4992032

[53] Lowe M. Structural organization of the Golgi apparatus. Curr Opin Cell Biol. 2011;23:85–93. 21071196 10.1016/j.ceb.2010.10.004

[54] Ayala I, Colanzi A. Structural organization and function of the golgi ribbon during cell division. Front Cell Dev Biol. 2022;10:925228. 35813197 10.3389/fcell.2022.925228 PMC9263219

[55] Egea G, Serra-Peinado C, Salcedo-Sicilia L, Gutiérrez-Martínez E. Actin acting at the Golgi. Histochem Cell Biol. 2013;140:347–60. 23807268 10.1007/s00418-013-1115-8

[56] Guet D, Mandal K, Pinot M, Hoffmann J, Abidine Y, Sigaut W, et al. Mechanical role of actin dynamics in the rheology of the Golgi complex and in Golgi-associated trafficking events. Curr Biol. 2014;24:1700–11. 25042587 10.1016/j.cub.2014.06.048

[57] Cremer T, Voortman LM, Bos E, van Elsland DM, ter Haar LR, Koning RI, et al. Vimentin intermediate filaments organize organellar architecture in response to ER stress. Preprint at 10.1101/2022.03.24.485587 2022.

[58] Kürekçi GK, Mangit EK, Koyunlar C, Unsal S, Saglam B, Ergin B, et al. Knockout of zebrafish desmin genes does not cause skeletal muscle degeneration but alters calcium flux. Sci Rep 2021; 11. 10.1038/s41598-021-86974-w. 10.1038/s41598-021-86974-w PMC8021586 33820917

[59] Fredrickson EK, Rosenbaum JC, Locke MN, Milac TI, Gardner RG. Exposed hydrophobicity is a key determinant of nuclear quality control degradation. Mol Biol Cell. 2011;22:2384–95. 21551067 10.1091/mbc.E11-03-0256 PMC3128539

[60] Soluble oligomerization provides a beneficial fitness effect on destabilizing mutations | PNAS. https://www.pnas.org/doi/full/10.1073/pnas.1118157109 (accessed 13 Aug2025). 10.1073/pnas.1118157109 PMC3323985 22411825

[61] van der Klooster ZJ, Sepehrkhouy S, Dooijes D, te Rijdt WP, Schuiringa FSAM, Lingeman J, et al. P62-positive aggregates are homogenously distributed in the myocardium and associated with the type of mutation in genetic cardiomyopathy. J Cell Mol Med. 2021;25:3160–6. 33605084 10.1111/jcmm.16388 PMC7957157

[62] Su H, Wang X. The ubiquitin-proteasome system in cardiac proteinopathy: a quality control perspective. Cardiovasc Res. 2010;85:253–62. 19696071 10.1093/cvr/cvp287 PMC2797449

[63] Ramos FJ, Chen SC, Garelick MG, Dai D-F, Liao C-Y, Schreiber KH, et al. Rapamycin reverses elevated mTORC1 signaling in lamin A/C–deficient mice, rescues cardiac and skeletal muscle function, and extends survival. Sci Transl Med 2012; 4. 10.1126/scitranslmed.3003802. 10.1126/scitranslmed.3003802 PMC3613228 22837538

[64] Rubinsztein DC, Nixon RA. Rapamycin induces autophagic flux in neurons. Proc Natl Acad Sci USA. 2010;107:E181. 21115811 10.1073/pnas.1014633107 PMC3000262

[65] Ju J-S, Varadhachary AS, Miller SE, Weihl CC. Quantitation of “autophagic flux” in mature skeletal muscle. Autophagy. 2010;6:929–35. 20657169 10.4161/auto.6.7.12785 PMC3039739

[66] Sukhareva KS, Smolina NA, Churkina AI, Kalugina KK, Zhuk SV, Khudiakov AA, et al. Desmin mutations impact the autophagy flux in C2C12 cell in mutation-specific manner. Cell Tissue Res. 2023;393:357–75. 37277577 10.1007/s00441-023-03790-6 PMC10406715

[67] Höllrigl A, Puz S, Al-Dubai H, Kim JU, Capetanaki Y, Weitzer G. Amino-terminally truncated desmin rescues fusion of des −/− myoblasts but negatively affects cardiomyogenesis and smooth muscle development. FEBS Lett. 2002;523:229–33. 12123837 10.1016/s0014-5793(02)02995-2

[68] Goldfarb LG, Dalakas MC. Tragedy in a heartbeat: malfunctioning desmin causes skeletal and cardiac muscle disease. J Clin Investig. 2009;119:1806–13. 19587455 10.1172/JCI38027 PMC2701871

[69] Srivastava K, Pandit B. Protocol for generation of single-gene knockout in hard-to-transfect THP1 cell lines using CRISPR/Cas9. Bio-Protoc 2025; 15. 10.21769/BioProtoc.5365. 10.21769/BioProtoc.5365 PMC12245624 40655415

[70] Footz T, Idrees F, Acharya M, Kozlowski K, Walter MA. Analysis of mutations of the PITX2 transcription factor found in patients with axenfeld-rieger syndrome. Investig Ophthalmol Vis Sci. 2009;50:2599–606. 19218601 10.1167/iovs.08-3251

[71] Schneider CA, Rasband WS, Eliceiri KW. NIH Image to ImageJ: 25 years of image analysis. Nat Methods. 2012;9:671–5. 22930834 10.1038/nmeth.2089 PMC5554542

