# Supplementary material for: Novel truncating Desmin mutation Arg150Stop disrupts structural integrity and cellular homeostasis by formation of persistent aggregate-like structure
Source: Cell Death Discov. 2026 Jun 5;12:332. doi: 10.1038/s41420-026-03184-x (PMC13458698; doi:10.1038/s41420-026-03184-x)
Supplement: Supplementary file 1 [file 41420_2026_3184_MOESM1_ESM.pdf]

**Primer ID**	**Primer sequence (5' – 3')**	**Regions Amplified (Product size in bp)**	**T_A (°C)**
DES-CL-WT-F1	CGGAATTC ATGTACCCATACGATGTTCCA GATTACGCTAGCCAGGCCTACTCGTCC	WT Desmin cDNA sequence (1440)	64
DES-CL-WT-R1	GCTCTAGATTAGAGCACTTCATGCTGCTG		
Des_SDM_RArg150Stop_FP	CGAGCCGACGTGAGTGGCCGAGC	Primers for Site-Directed Mutagenesis	65
Des_SDM_Arg150Stop_RP	CGGCCCTTGAGCCGGTTCAC		
pCI-T7-FP	TAATACGACTCACTATAGGG	pCI specific forward primer T7-promoter for sanger sequencing	
EBV-RP	GTGGTTTGTCCAAACTCATC	pCI specific reverse primer for sanger sequencing	
Des_sgRNA Forward	CACCGcgctcgccgccgaagtgaac	Lentivirus mediated Desmin R150X Mutant Cell Line Generation	
Des_sgRNA Reverse	AAACgttcacttcggcggcgagcgC	Lentivirus mediated Desmin R150X Mutant Cell Line Generation	

Table S1: List of primers used

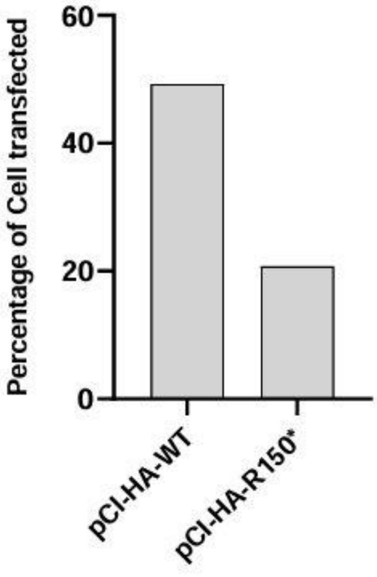

Figure S1: Transfection Efficiency of pCI-HA-WT and pCI-HA-R150X using Neon Transfection System (Invitrogen).

Plasmid	
psPAX2 (Addgene Plasmid #12260)	2nd generation lentiviral packaging plasmid
pMD2.G(Addgene Plasmid #12259)	VSV-G envelope expressing plasmid
lentiCRISPR v2<br>(Addgene Plasmid #52961)	Lentiviral transfer plasmid, also known as pLentiCRISPR v2, for CRISPR/Cas9 genome editing of mammalian cells with a single guide RNA (sgRNA)

Table S2: List of plasmids used for Lentivirus mediated Desmin R150X Mutant Cell Line Generation

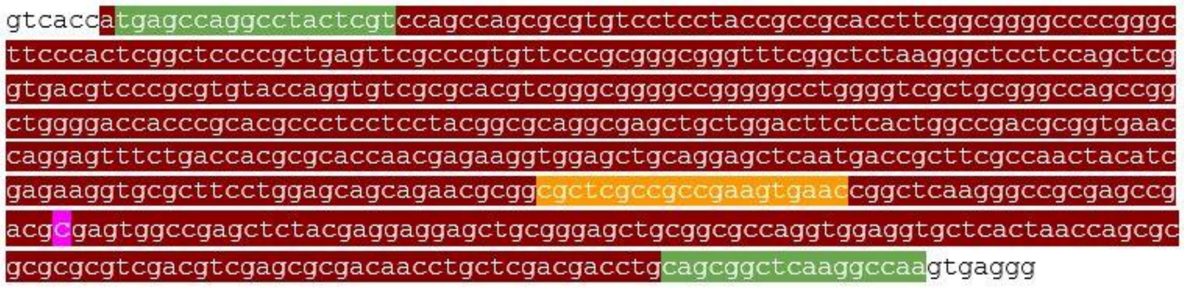

Figure S2: Sequence of exon 1 of the DES gene showing the c.448C nucleotide (highlighted in purple) and the corresponding sgRNA binding site (highlighted in orange).

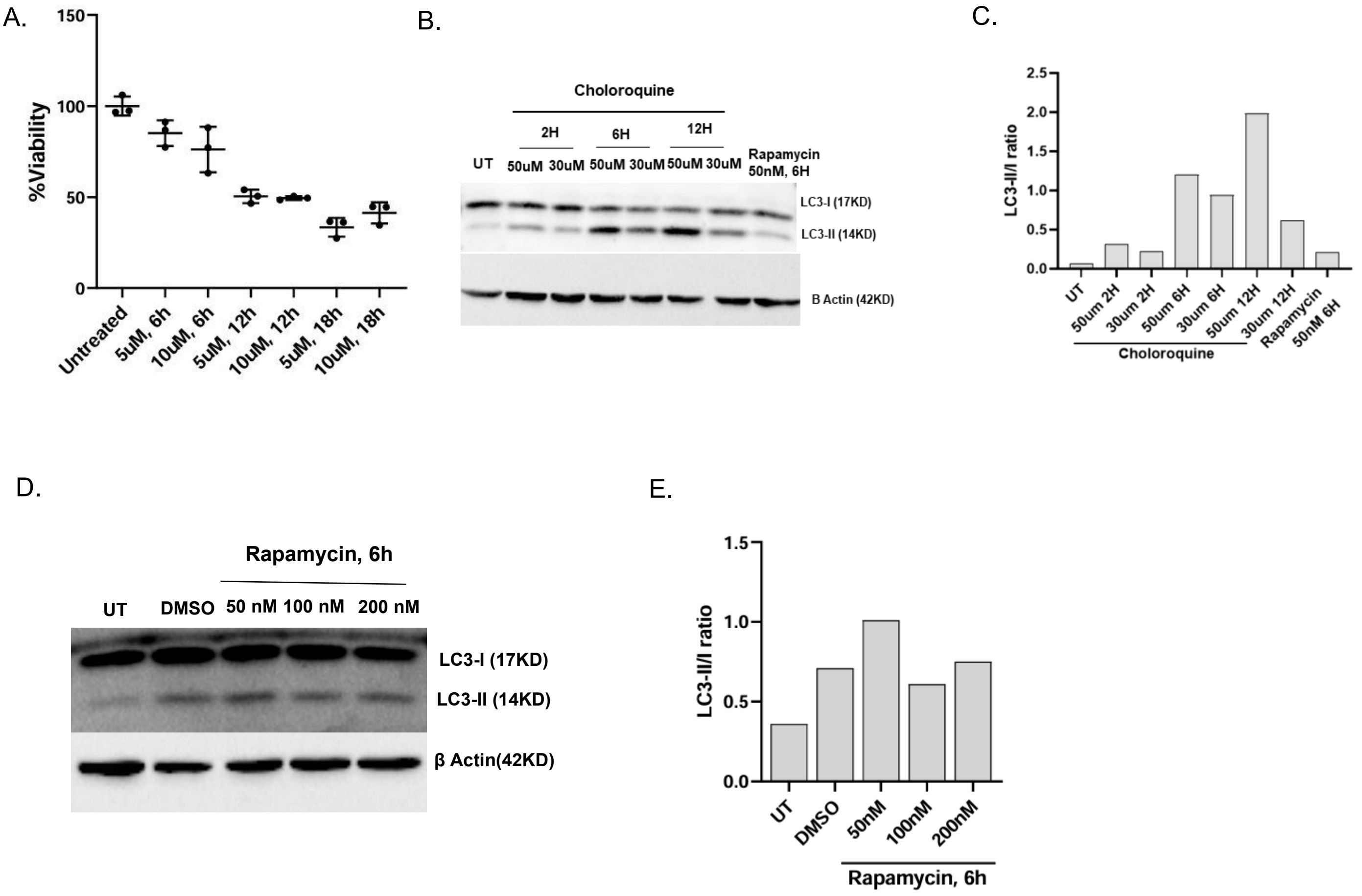

Figure S3: (A)The optimal dose and treatment duration of MG132 were standardized based on cell viability measurements using the MTT assay. (B, C, D and E) Standardization of the optimal concentration of Chloroquine and Rapamycin by accessing LCII/LC3-I ratio via immunoblotting.

Antibody used in Western Blot	Identifier	Dilution
HA-Probe (F-7), Mouse monoclonal IgG2a (Cat no: sc-7392)	Santa Cruz Biotechnology	1:1000
Rabbit Anti-Desmin antibody, Source no- 21190 (Cat no: D8281-1ML)	Sigma-Aldrich	1:1500
polyclonal Rabbit LC3B (PM-036)	MBL International	1:1000
ACTB; Mouse monoclonal (C4) (cat no: sc47778)	Santa Cruz Biotechnology	1:1000
Goat Anti-Rabbit IgG H&L (cat no: ab97051)	Abcam	1:10,000
Goat Anti-Mouse IgG H&L (cat no: ab205719)	Abcam	1:10,000

Antibody used in Immunofluorescence	Identifier	Dilution
HA-Probe (F-7), Mouse monoclonal IgG2a (Cat no: sc-7392)	Santa Cruz Biotechnology	1:200
HA-Tag (C29F4) Rabbit mAb (Cat: 3724S)	Cell Signalling Technology	1:400
Rabbit Anti-Desmin antibody, Source no- 21190 (Cat no: D8281-1ML)	Sigma-Aldrich	1:20
mouse monoclonal anti-alpha smooth muscle actin antibody (cat no: ab7817)	Abcam	1:200
rabbit polyclonal anti-PLS1 antibody (cat no: ab236976)	Abcam	1:200
rabbit polyclonal to anti-alpha actinin/ACTN1 antibody (cat no: ab155480)	Abcam	1:50
Purified Mouse anti-GM130 (Catno: 610822)	BD Transduction Laboratories™	1:200
Rabbit polyclonal GCC2	Altas Antibodies]	1:500
Purified Mouse Anti-Human p230 trans Golgi (Cat no: 611280)	BD Transduction Laboratories™	1:1000
mouse Monoclonal Calnexin (AF18) (Cat no: 23954)	Santa Cruz Biotechnology	1:200
mouse monoclonal anti-SQSTM1 / p62 antibody [2C11] (Cat no: ab56416)	Abcam	1:200
MitoTracker™ Red CMXRos (cat no: 9082)	Cell Signalling Technology	50nM

Table S3: List of antibodies used for western blot

Table S4: List of antibodies used for Immunofluroscence

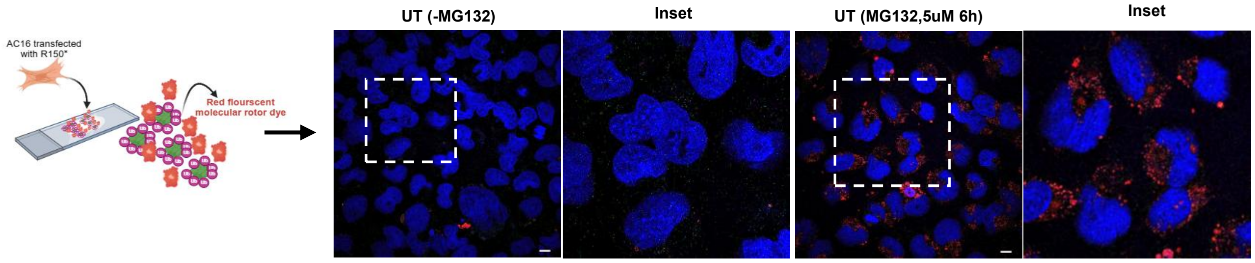

Figure S4: Validation of aggresomal fluorescence detection using control treatments. UT (-MG132) is negative control and UT (MG132, 5uM, 6h) is positive control.

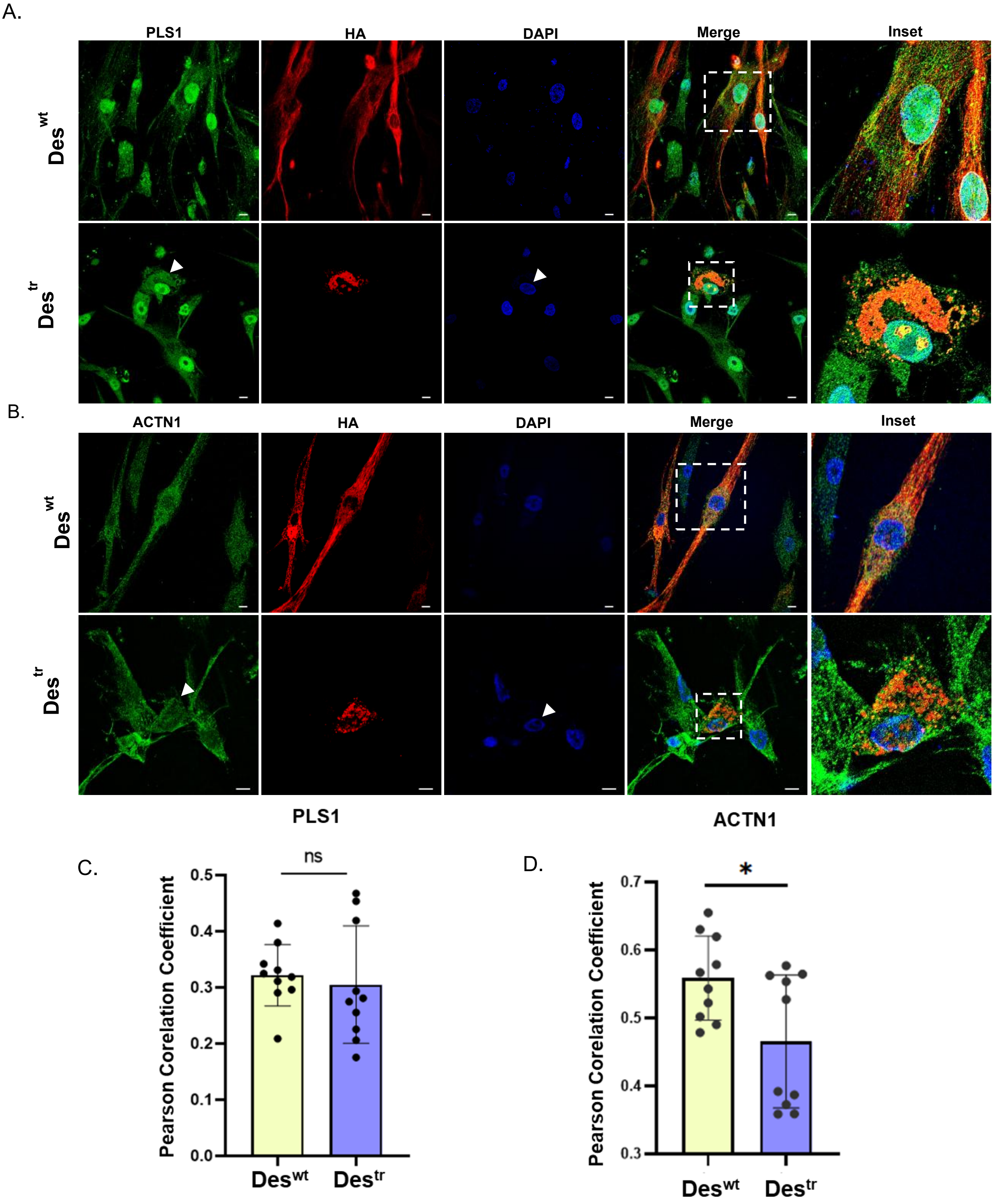

Figure S5: The distribution of Actin-binding proteins was perturbed in cells expressing truncated Arg150stop desmin mutant depicted that Des^{tr} affected the morphology of skeletal muscle cells.

A,B. Des^{wt} and Des^{tr} transfected cells expressing HA tagged desmin (red) and co-stained for endogenous PLS1 and ACTN1 respectively (green).

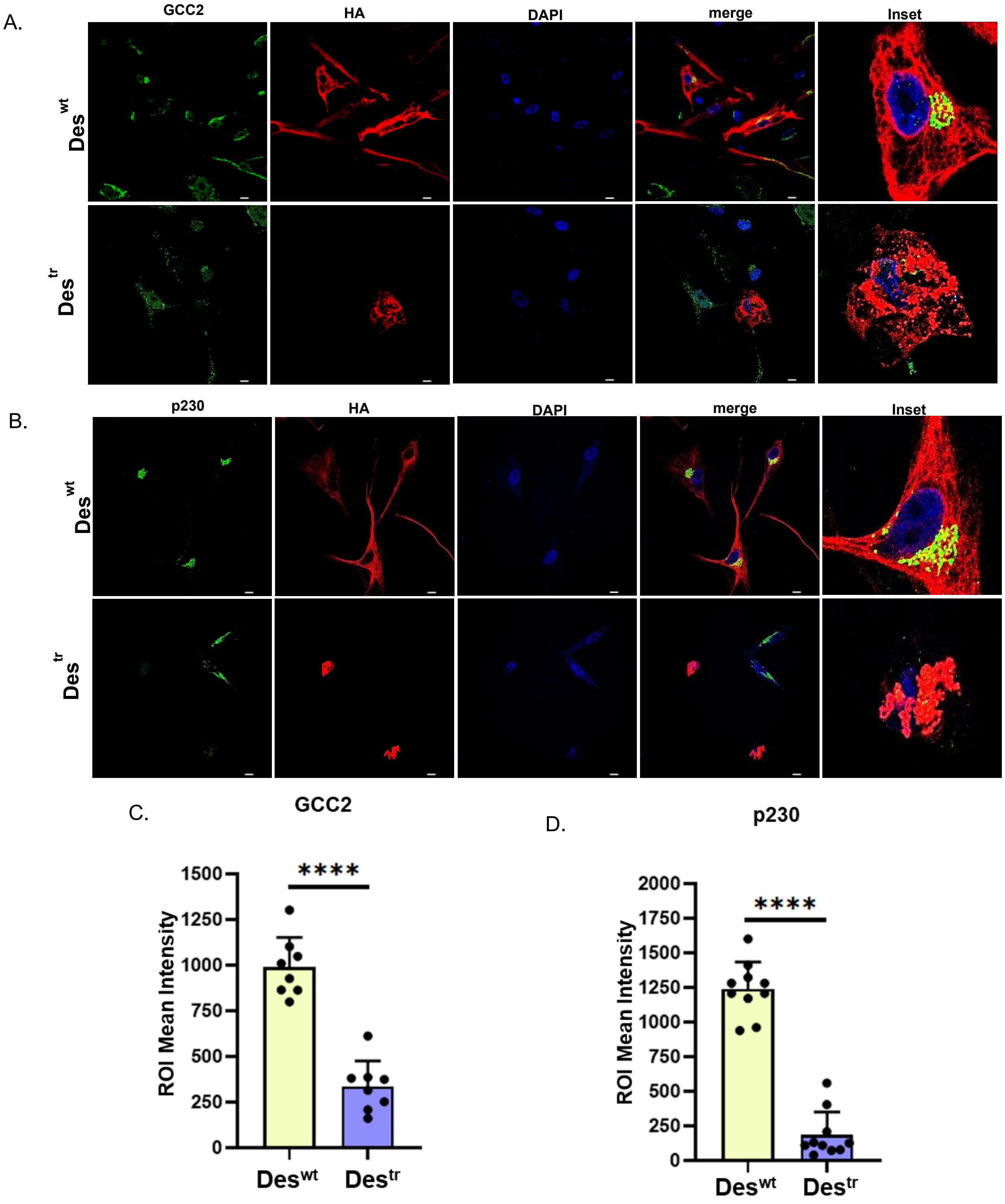

Figure S6: The expression of trans-golgi markers were also perturbed in cells expressing truncated Arg150stop desmin mutant

A,B. Des^{wt} and Des^{tr} transfected cells expressing HA tagged desmin (red) and co-stained for endogenous GCC2 and p230 respectively (green)

C,D. ROI mean Intensity of transiently expressed desmin and endogenous GCC2 and p230 respectively

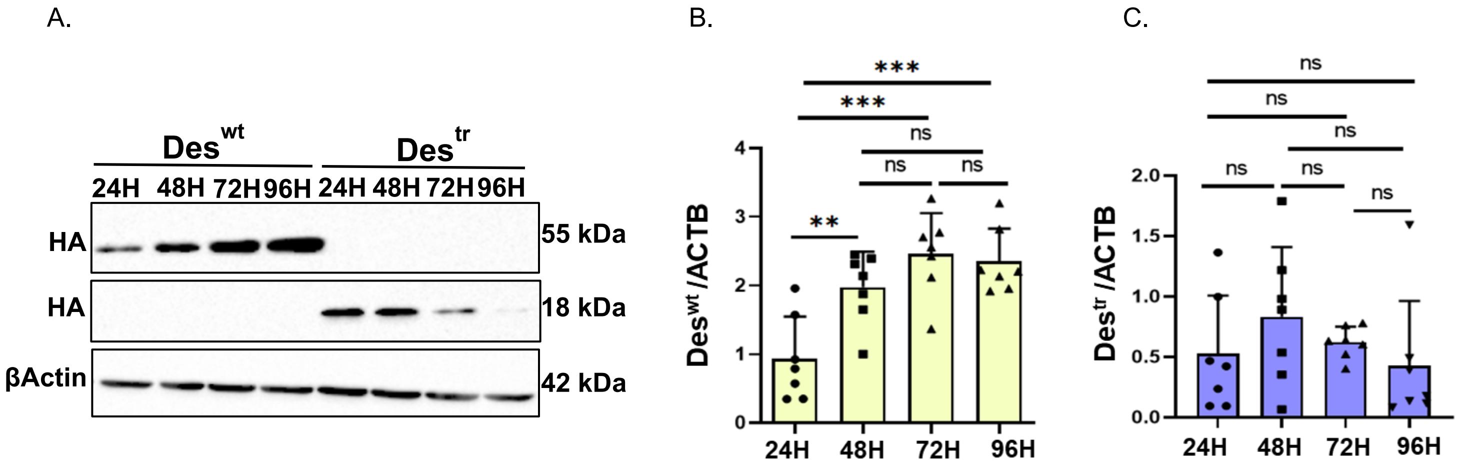

Figure S7: Western blot showing expression of wild-type and truncatedArg150stop desmin mutant under basal conditions.

(A) Immunoblot showing the expression of wild-type and truncated desmin

(B) Densitometric analysis of wild-type desmin at four different time-point depicts a gradual increase in the overexpression of Des^{wt} over time (n=7, **p=0.0050[24v/s48], ***p=0.0005[24v/s72], ***p=0.0004[24v/s96]).

(C) Densitometric analysis of truncated desmin at four different time-point depicts no gradual change in the overexpression of Des^{tr} over time (n=7).

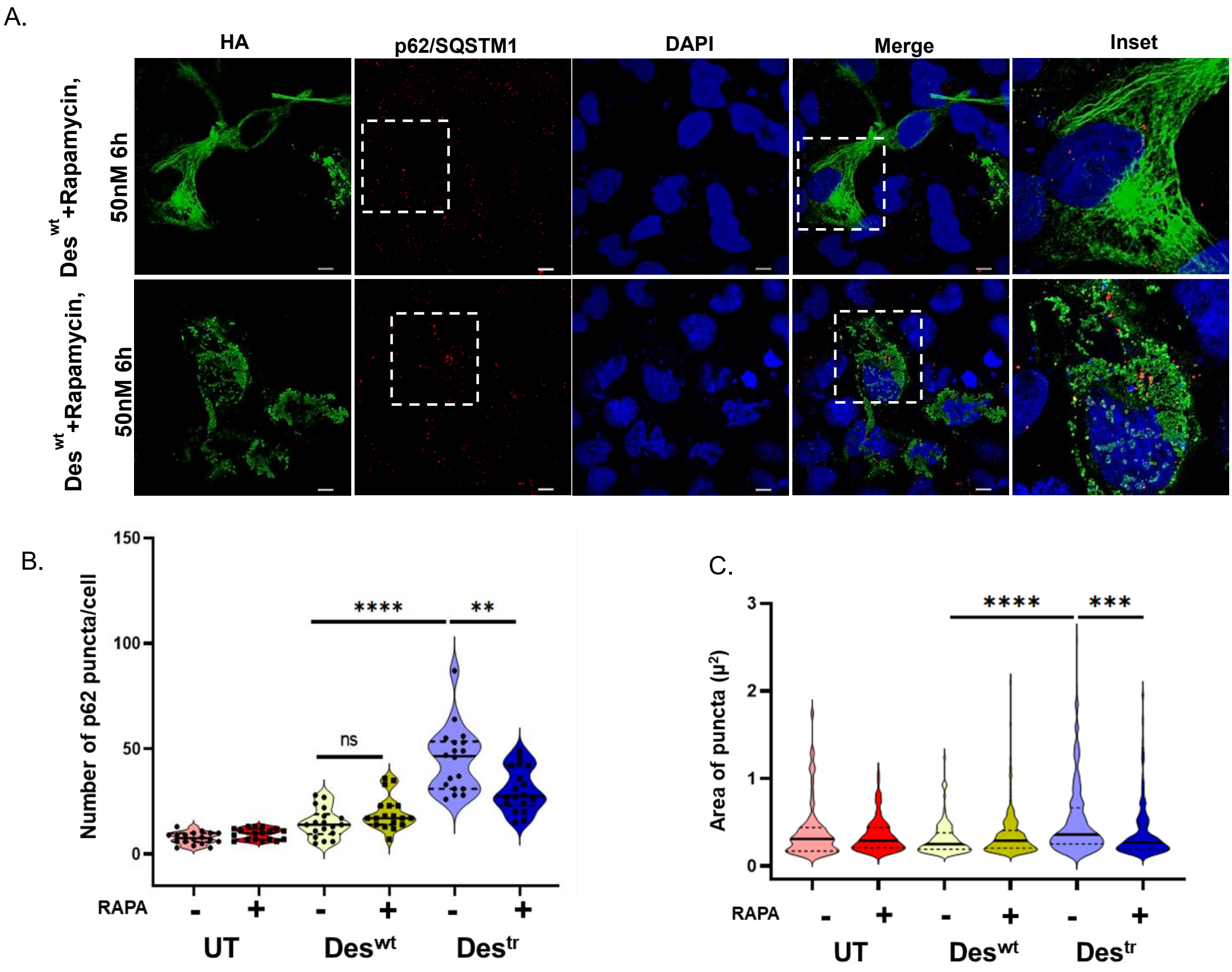

Figure S8: Immunofluorescence data showing p62 puncta number and area diminishes upon Rapamycin (50nm, 6h) treatment in truncated Arg150stop desmin mutant

A. Confocal microscopy images (60X) of cells transfected with Des^{wt} and Des^{tr}, shows wild-type and mutant R150X desmin expression in presence of rapamycin treatment.
B. Quantification of p62 puncta number per cell, showing decreased p62 accumulation in truncated Arg150stop desmin mutant transfected cells after rapamycin treatment (n=18, **p=0.0025).
C. Quantification of p62 puncta area, showing reduction in size after rapamycin treatment (***p=0.0006).

RAPA-Rapamycin
